# Environment-mediated interactions cause an externalized and collective memory in bacteria

**DOI:** 10.1093/ismejo/wraf173

**Published:** 2025-08-11

**Authors:** Shubham Gajrani, Xiaozhou Ye, Christoph Ratzke

**Affiliations:** Interfaculty Institute for Microbiology and Infection Medicine Tübingen (IMIT), Cluster of Excellence EXC 2124 “Controlling Microbes to Fight Infections” (CMFI), University of Tübingen, Calwerstr. 7/1, 72076 Tübingen, Germany; Interfaculty Institute for Microbiology and Infection Medicine Tübingen (IMIT), Cluster of Excellence EXC 2124 “Controlling Microbes to Fight Infections” (CMFI), University of Tübingen, Calwerstr. 7/1, 72076 Tübingen, Germany; Interfaculty Institute for Microbiology and Infection Medicine Tübingen (IMIT), Cluster of Excellence EXC 2124 “Controlling Microbes to Fight Infections” (CMFI), University of Tübingen, Calwerstr. 7/1, 72076 Tübingen, Germany

**Keywords:** community assembly, externalized memory, microbial interactions, collective memory, microbial communities, niche construction, multistability, complex systems

## Abstract

Bacteria usually live in complex communities interacting with many other microbial species. These interactions determine who can persist in a community and how the overall community forms and functions. Bacteria often exert interactions by chemically changing the environment, like taking up nutrients or producing toxins. These environmental changes can persist over time. We show here that such lasting environmental changes can cause a “memory effect” where current growth conditions alter interaction outcomes in the future. This memory is only stored in the environment and not inside bacterial cells. Only the collective effort of many bacteria can build up this memory, making it an emergent property of bacterial populations. This externalized and collective memory can also impact the assembly of more complex communities and lead to different final compositions depending on the system’s past. Overall, we show that to understand interaction outcomes fully, we have to consider not only the interacting species and abiotic conditions but also the system’s history.

## Introduction

The ability to form memory, that is, storing information of the past that impacts future events, is a central property of biological systems across many scales. On the molecular level, information can be stored, e.g. by modification of the genome [1, 2], positive feedback loops of gene expression [3], or inheritance of long-lived proteins [4]. Although some of these memory effects may increase an organism’s fitness, others may just be by-products of the complexity of biological systems [5].

Whereas memory is often stored inside an organism, this does not have to be the case necessarily. On the ecological scale, changes of the environment by organisms have been described as a possible source of memory impacting the future of ecosystems [6, 7]. In particular, organisms can alter ecosystems (ecosystem engineering, niche construction [8]), and these changes can have lasting effects on other organisms even after the causal species disappeared (legacy effect) [9, 10]. For example, soil microbiomes were shown to be more impacted by the past than current vegetation [11]. Bacteria can alter soil structures and change water infiltration, potentially impacting ecosystems over long times [12], and chemical modifications by bacteria in the gut can protect from pathogens [13]. An overview of ecological effects that exhibit some form of memory is provided (Supplementary Table 1).

**Figure 1:**
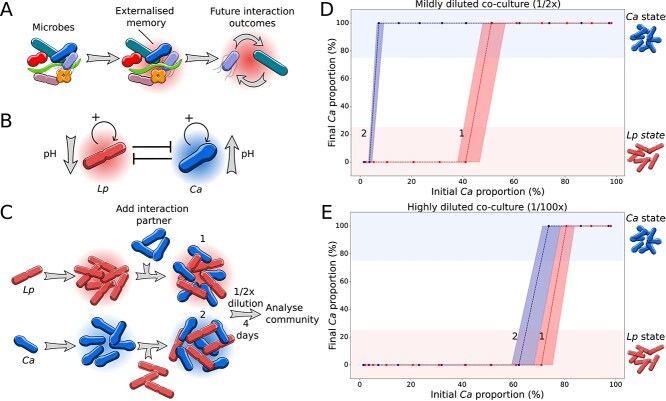
Microbial interaction outcomes depend on the system’s past. (A) Microbial communities can chemically change the environment, and this chemical modification depends on the bacterial growth conditions. If this chemical change persists over time, future interactions may be affected by the chemical change, and thus, events in the past can impact future interaction outcomes. (B) In our two-species model system, *Lp* decreases the pH of the medium, whereas *Ca* increases the pH of the medium, thus inhibiting each other. (C) Adding *Ca* to an established culture of *Lp* and vice versa can result in different interaction outcomes. When a species interacts in its own memory, it has a higher chance of outcompeting the interaction partner. However, the effect only occurs at sufficiently high population densities (D) (*n* = 3) and disappears upon dilution (E) (*n* = 3). Dashed lines in (D) and (E) show median, and shaded areas represent minimum and maximum values based on >50% *Ca* relative abundance.

Bacteria are strong niche constructors, known to alter their environment by secreting many metabolites, from simplebyproducts of energy metabolism to complex toxins [14]. The metabolites often directly impact their own and other bacterial growth and thus can drive microbial interactions [15, 16]. Which metabolites the bacteria secrete depend strongly on the microbial metabolism and, therefore, the environmental conditions the bacteria face, such as temperature [17], oxygen levels [18], or toxin concentrations [19]. This situation raises the question of whether bacteria can show a type of externalized memory where the current conditions lead to the generation of different chemical changes in the environment that further impact the growth and interactions of bacteria in the future (Fig. 1A). Because a single microbe has a minimal potential to change the environment [20, 21], we hypothesize that the formation of such externalized memory should require the collective action of a large number of bacteria. Microbial communities could establish a memory by collectively secreting metabolites, and thus performing ecosystem engineering, despite no memory being present in the individual cell (Fig. 1A). Such a memory would be an emergent collective phenomenon.

We show in the following that collective, externalized memory can indeed originate from relatively simple features of microbial interactions and impact microbial interactions based on past events. Such a memory can lead to surprising reactions of a bacterial community to temperature or oxygen. Finally, we could show that externalized memory can alter the assembly of complex communities.

### Materials and methods

#### Media and buffers

Nutrient media (NM) was prepared with 10 g/l yeast extract (J23547, Thermo Scientific, Kandel, Germany) and 10 g/l soytone (91079-46-8, Merck Millipore, Darmstadt, Germany) mixed in distilled water and pH adjusted to 7. NM was then autoclaved at 121°C for 20 min at 103 kPa.

Base media (BM) was prepared by mixing 1 g/l yeast extract, 1 g/l soytone, and 10 mM NaH_2_PO_4_ (28011.291, VWR, Darmstadt, Germany) in distilled water, and the pH was adjusted to 6.5 (8172BNWP, 2 115 001, Thermo Scientific, Braunschweig, Germany). BM was autoclaved as described for NM above. Before usage, 0.1 mM CaCl_2_ (A4689.0250, PanReac AppliChem, Darmstadt, Germany), 2 mM MgSO_4_ (11596, Thermo Scientific, Kandel, Germany), 4 mg/l NiSO_4_, and 50 mg/l MnCl_2_ were added.

Supplemented base media (SBM) was prepared by adding 1% w/v D-glucose (0188-2.5KG, VWR, Darmstadt, Germany) and 0.8% w/v urea (U/0500/53, Fisher Scientific AG, Reinach, Switzerland) to base media and vacuum filtering through a 0.2 μm filtration unit (Filtropur V50 83.3941.001, Sarstedt, Nümbrecht, Germany).

NM Agar plates were prepared by dissolving 10 g/l yeast extract, 10 g/l peptone, 10 mM NaH_2_PO_4_, and 2.5% agar (5210.2 Carl Roth, Karlsruhe, Germany) in 980 ml water. The mixture was autoclaved, and 50 ml solution was poured into each 150 × 200 mm Petri dish (82.1184.500 Sarstedt, Nümbrecht, Germany). Before usage, plates were dried at room temperature for 3 days. For pH-selective NM Agar plates, the pH of the mixture was adjusted to either 5 or 10 before autoclaving, and after autoclaving, 0.02% sterile glucose was added to the mixture upon cooling.

To make M9-K minimal media, 43 mM NaCl (12 314, Alfa Aesar, Thermo Scientific, Kandel, Germany), 54 mM KCl (1.04936.1000, Supelco, Merck SA, Darmstadt, Germany), 93 mM NH_4_Cl (21236.267, VWR, Darmstadt, Germany), and 10 mM NaH_2_PO_4_ were dissolved in distilled water, pH was adjusted to 7, filled up to 1 l with distilled water, and autoclaved as described for NM media above. After cooling down, the sterile addition of 1 ml/l 1000× trace metals mix (Teknova, Hollister, CA, USA T1001), 2 mM MgSO_4,_ and 0.1 mM CaCl_2_ was carried out.

Tryptic soy broth (TSB) was prepared by mixing 17 g/l tryptone (95039-1KG-F Sigma Aldrich, Merck SA, Darmstadt, Germany), 3 g/l soytone, 5 g/l NaCl, 2.5 g/L K_2_HPO_4_ (191 431, MP Biomedicals, Eschwege, Germany)_,_ and 2.5 g/l D-glucose in 1 l distilled water. The medium was sterilized using vacuum filtration through a 0.2 μm filtration unit.

Phosphate-buffered saline (PBS) + 0.4% Tween-80 was prepared by diluting 8 g/l NaCl, 200 mg/l KCl, 10 mM Na_2_HPO_4_ (30435-1KG, Honeywell, Seelze, Germany), 1.8 mM KH_2_PO_4_ (26923.298, VWR, Darmstadt, Germany), and 0.4% v/v Tween-80 (8.22187.0500 Sigma-Aldrich, Merck SA, Darmstadt, Germany) in distilled water, followed by sterilization with autoclave as described for NM above.

#### Bacterial strains

Lactobacillus plantarum *(*referred to as *Lp*) is a Gram-positive, nonmotile, lactic acid–producing bacterium acquired from the American Type Culture Collection (ATCC8014). Corynebacterium ammoniagenes (referred to as *Ca*) is a Gram-positive aerobic bacterium and was also obtained from the American Type Culture Collection (ATCC 6871).

Gut strains were isolated from C. elegans*,* from Massachusetts, USA [22], or from northern Germany (*An*) [23]. Eight strains (*Bt, Bf, Rb, Pv, Pp, Sm, Rq, An*) were selected to ensure different colony sizes, colors, and morphology—to distinguish them upon colony counting as described below, as well as ensuring phylogenetic diversity (Supplementary Figs 12B and 13). Bacteria were streaked on NM agar plates, and colonies were allowed to form by incubation at 30°C for 24 h. The plates were subsequently stored at 4°C for up to 1 week. Single colonies from these plates were used to start the experiments described below.

#### Two-species memory assay

Single colonies of *Lp* and *Ca* were grown in 5 ml NM overnight in 50 ml tubes (62.547.254, Sarstedt, Nümbrecht, Germany) at 30°C shaking at 225 rpm (Innova 2000, New Brunswick Scientific, Nürtingen, Germany). The lids were left open for one-eighth of the turn and fixed with tape. After 18 h, bacterial cultures were centrifuged at 2451 rcf for 3 min and resuspended in 5 ml SBM. Resuspended cultures were 1/100× diluted into 2 × 5 ml fresh SBM cultures for each species in 50 ml tubes for incubation at 30°C on shaking for 24 h. The following day, all 5 ml cultures from the same species were pooled and split into two tubes—“washed” cultures and “unwashed” cultures.

To make washed cultures, bacterial cultures were centrifuged at 2451 rcf for 3 min (Centrifuge 5810, Eppendorf, Hamburg, Germany), and the supernatant was taken out and filtered into fresh 50 ml tubes using a 0.2 μm syringe filter (83.1826.001, Sarstedt, Nümbrecht, Germany). Pelleted bacteria were resuspended and washed twice with fresh SBM. OD_600_ was taken using Implen OD_600_ reader for washed and unwashed bacterial cultures. OD_600_ = 1 cultures were prepared for washed samples using fresh SBM and with respective supernatants for unwashed samples for each species. Dilution rows for OD_600_ = 1 cultures were prepared with PBS + 0.4% Tween and plated with 5 μl droplets using Viaflo 96 for initial CFU counts on pH 5 NM plates for *Lp* and pH 10 NM plates for Ca.

*Lp* unwashed was mixed with *Ca* washed culture and vice versa in increasing percentages from 2% to 98%. Mixed cultures were diluted with SBM with ratios of 1/2×, 1/6×, 1/20×, and 1/100× in 96 deep well plates (786 201, Greiner Bio-One Frickenhausen, Germany). The plate sealed with breathable sterile rayon film (VWR 391-1262) was incubated on shaking at 1250 rpm on a platform shaker (Titramax 100, Heidolph Instruments GmbH, Schwabach, Germany) at 30°C. 1/2× dilutions with fresh SBM were carried out daily for 4 days. Dilution plates were made with 1/10× dilution in PBS + 0.4% Tween in 96-well microtest plates (82.1581.001, Sarstedt, Nümbrecht, Germany), followed by droplet plating of 96-well plates on pH-selective NM Agar plates with 5 μl droplets using Viaflo 96. pH-selective NM agar plates were incubated at 30°C for 48 h before CFU counting using a stereo microscope (TL3000 Ergo, Leica Microsystems, Wetzlar, Germany). From observed CFU/ml values, actual *Lp*/*Ca* ratios, and percentages were calculated.

In order to check the effect of “reduced nutrients” on externalized and collective memory, constituents of SBM were diluted by a factor of two (referred to as 1/2× SBM) and five (referred to as 1/5× SBM). Using three minimal media (SBM, 1/2× SBM, 1/5× SBM), a similar experimental protocol was followed as described above.

#### Two-species memory assay under different growth conditions

**Figure 2:**
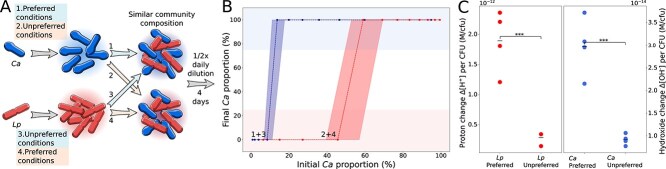
Past growth conditions impact microbial interactions through externalized memory (A) *Ca* grown in preferred conditions was mixed with *Lp* grown in unpreferred conditions and vice versa as described in the methods. (B) Externalized memory formed in preferred conditions enables *Ca* to outcompete *Lp* at lower relative abundances as compared to when *Ca* memory is formed under unpreferred conditions. The effect diminishes with increasing dilution of memory with fresh media (Supplementary Fig. 5). Dashed lines show again the median curves and shaded areas minimal and maximal curves (*n* = 3). (C) To study how different growth conditions affect environmental changes, both bacteria were grown in their corresponding preferred and unpreferred conditions. Preferred conditions (*Lp:* 35°C and anoxic, *Ca:* 35°C and oxic, Supplementary Fig. 4A) were marked by greater proton change in case of *Lp* (two-tailed Student’s *t*-test, *P-*value = .0008, normal distribution of data tested by Shapiro–Wilk test *P-*value = .584) and greater hydroxide change per CFU in case of *Ca* (two-tailed Student’s *t*-test, *P-*value = .0007, normal distribution of data tested by Shapiro–Wilk test *P-*value = .104) as compared to that of unpreferred conditions. Data points represent four biological replicates.

For studying the effect of temperature on externalized memory, two SBM cultures were prepared for each species (after primary culture in NM, as described above)—one grown in preferable conditions and the other grown in unpreferable conditions (Fig. 2A). Depending on the different physiological requirements of the bacteria, 2 × 50 ml tubes (per condition per species) were either covered with breathable rayon film (Ca_Pref_ and Lp_Unpref_) or completely closed (Lp_Pref_ and Ca_Unpref_) during incubation at either 35°C (Ca_Pref_ and Lp_Pref_) or at 18°C (Ca_Unpref_ and Lp_Unpref_) on shaking at 225 rpm for 24 h.

The following day, bacterial cultures of the same species and conditions were pooled and centrifuged at 2451 rcf for 3 min, and half of the supernatant per tube was taken out and filtered into fresh 50 ml tubes using syringe filters. Optical density reading was taken, and using their respective filtered supernatants, OD_600_ = 1 was adjusted for both species at both conditions. Dilution plating for initial CFU counts was performed as described above.

Ca_Pref_ was mixed with Lp_Unpref_, and Ca_Unpref_ was mixed with Lp_Pref_ in increasing percentages from 2% to 98%, and mixed cultures were diluted with SBM with ratios: 1/2×, 1/6×, 1/20×, and 1/100× in 96 deep well plates. The following steps were similar to those outlined above.

#### Mathematical model, parameter selection, and simulation

**Figure 3:**
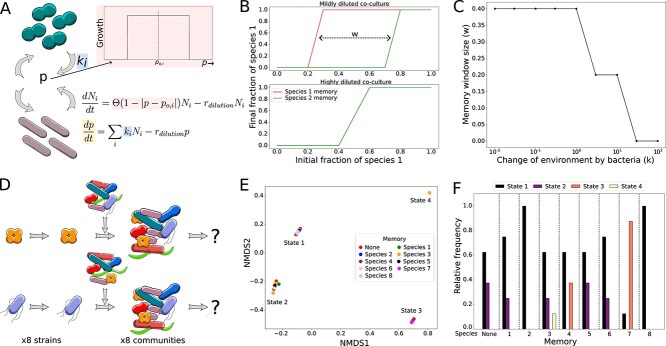
A simple model can recapitulate the observed memory effect and suggests that it affects community assembly. (A) In order to simulate microbial interactions in the presence of collective memory, the shown equations were used, where the growth rate of species *N_i_* is determined using the Heaviside step function, θ, of the difference between environmental variable *p* and optimal environmental variable *p_o,i_*. The rate of change in the environmental variable is governed by *k_i_* and the dilution rate *r_dilution_*. This system ensures maximum growth rate of the species when the environmental variable *p* is close to the respective optimum value for the species, as illustrated in the *p* vs growth plot. The term r_dil_*p_in_ is absent in equation (2) as *p_in_* is set to 0. (B) Simulating the equations for two mutually excluding species and one environmental variable for different initial mixing ratios reveals that a species has a higher chance to outcompete the other species in the presence of its own memory, recapitulating the memory effect observed experimentally. Window size (w) refers to the difference in the initial fraction of Species 1 (for different memories added) at half-height (i.e. final fraction of species 1 = 0.5). (C) A faster change of the environment by the bacteria *k* (*k_1_* = *k*, *k_2_* = *−k*) removes the memory effect. The window size shown on the *y*-axis corresponds to the difference between the two curves for both species’ memory as depicted in (B). (D) Extending the complexity to eight species and three environmental variables, we simulated the assembly of several multispecies communities in different memories. (E) Eight such communities were simulated in eight different memories, as well as the absence of memory (depicted by “None”). The communities end up in four distinct states as shown in an NMDS plot (Supplementary Fig. 7B). (F) Certain species’ memories can cause the same initial communities to end up in different final states as depicted in (E).

Simulations were performed in Python 3.9.7 based on the mathematical model of the system dynamics (Fig. 3A). To recapitulate the two-species system, the number of interacting species *N_i_* was set to *i* = 2. Both species influence one environmental variable *p*, which can be positive or negative*. k* and *p_o_* were set to [−1, 1] and [−3, 3], corresponding to two species that change the environmental variable *p* in opposite directions (Fig. 1B) at an equal absolute rate k_1/2_. Species were grown individually with *p_initial* = 0, *N_i__initial* = 1*,* and *r_dilution_* = 0.1. *p_initial* is the initial value of *p* in the media and therefore also the value of added media. The steady-state mean *p_stst_* values were recorded to be utilized as *p_initial* in the presence of externalized memory in the following. To simulate species interactions, the initial *N_1_* values were set to {0.001, 0.01, 0.1, 0.2, … 0.999}, and correspondingly, initial *N_2_* values were set to 1-*N_1_* (see x-axis of Fig. 3B)*.* The initial *N_i_*, as well as *p_initial*, were subjected to various dilutions with dilution factors of {1, 0.3} (Fig. 3B). To obtain the population densities and environmental variables over time, the differential equations (Fig. 3A) were integrated with the solve_ivp module in Python. The obtained steady-state values are plotted (Fig. 3B). In an effort to ascertain the role of *k_i_* in the memory window size, simulations were performed over multiple values of *k* ∈ {0.01, 0.03, 0.1, 0.3, 1, 3, 10, 30, 100} such that values of *k_1/2_* are [*−k, k*] for two species in one simulation run (Fig. 3C).

To theoretically explore the assembly of more complex communities, we simulated eight species influencing three environmental variables based on extended versions of equations (1 and 2) (Fig. 3).


(1) \begin{equation*} \frac{d{N}_i}{dt}={N}_i\prod \limits_j\varTheta -\mid{p}_{j-}{p}_{o,i,j}\mid -{r}_{dilution}{N}_i \end{equation*}

(2) \begin{equation*} \frac{d_{pj}}{dt}=\sum \limits_i{k}_{i,j}{N}_i-{r}_{\mathrm{dilution}}{p}_j \end{equation*}

The values of *k_i_* and *p_o,i,j_*, being the optimal value of environmental variable *j* for species *i*, were chosen from uniform distributions using the numpy random. Rand function, with *p_o,i,j_* values ranging from −2 to +3 and *k_i_* values ranging from −10^−3^ to 10^−3^. *P_o,i,j_* values for each species i and environmental variable *j* were computed using numpy.random.rand function around asymmetric distribution [−2 to 3] because symmetric choice of values can lead to opposite changes nearly compensate each other, and the system might take much longer to reach a steady state. To obtain each species’ memory, single species growth was simulated with all *p*_initial*_i_* set to zero, *N*_initial*_i_* = 10, *r_dilution_* = 0.1, and their respective *k_i_* and *p_o,i,j_* values, and steady-state *p_stst,i_* values for each species *i* were acquired. Then, eight different communities were assembled with different initial abundances of the same eight species (with distinctive *k_i_* and *p_o,i,j_* values) in the memory of a single species. The different initial abundances *N*_initial*_i_* for each species *i* in the communities were randomly selected from {0.1,1,10} (Supplementary Figs 7A and 11A). *p_initial_i_* was set to *p_stst,i_* values resulting from single species *i* growth. In the absence of memory, *p_initial_i_* were all set zero. The Python function solve_ivp was utilized to perform simulations on equations (1 and 2) (Fig. 3A). The final *N_i_* values were used to calculate Bray–Curtis dissimilarities, and, based on that, NMDS was performed using sklearn.manifold.MDS module. The outcomes were plotted using the matplotlib module.

#### Community assembly and determination of final community composition

For each of the eight C. elegans gut strains (see above and Supplementary Fig. 13), precultures (5 ml) were grown in 50 ml tubes in tryptic soy broth (TSB) overnight at 30°C on shaking at 225 rpm. The following day, TSB cultures were washed in M9-K + 0.05% glucose and 1/100× inoculated into TSB as well as M9-K + 0.05% glucose cultures for each species in volumes of 5 ml at 30°C for 24 h at shaking. The next day, M9-K + 0.05% glucose cultures were centrifuged, and the supernatant was filtered using syringe filters.

Communities with different initial abundances (Supplementary Fig. 12A) were mixed with Python script–directed Echo 525 liquid handling system using 384PP plates (001-14 622, Beckman Coulter, Krefeld, Germany) as source and destination plates in three replicates. After shaking at 1800 rpm for 2 min, 6.67 μl of mixed community cultures was added to respective wells with 93.3 μl of each species filtered supernatants and 100 μl of fresh M9 + 0.05% glucose per well in a 96-deep well plate and sealed with breathable sterile rayon film. As a control, the mixed communities were also grown without the addition of filtered supernatant. Daily dilution of 1/30x was performed into fresh M9 + 0.05% glucose for 12 days, and final communities were plated on NM agar plates. NM agar plates were incubated at 30°C for 48 h before CFU identification and counting. To ensure the stability of the final states, plating on NM agar plates was also performed on Day 5 and Day 10 (Supplementary Figs 14 and 16).

#### Data analysis

All the plots were plotted using the matplotlib module in Python 3.9.7, whereas the illustrations were created with Inkscape. Non-metric multidimensional scaling and hierarchical clustering were performed using the scipy module in Python 3.9.7, whereas ANOSIM was performed using the scikit-bio module in Python 3.9. For the assignment of clusters, the fcluster function from scipy.cluster.hierarchy was used with the “distance” criterion and a cutoff at height 0.92. The cutoff was chosen based on observing roughly two states in the raw data (Supplementary Fig. 16).

## Results

### Microbial interaction outcomes are shaped by the system’s past

To explore the idea of collective and externalized memory in bacteria, we started with a simple model system consisting of the two bacteria, L*.* plantarum (*Lp*) and C*.* ammoniagenes (*Ca*)*.* We used this model system in the past to study microbial interactions. In media that contain glucose and urea as major carbon and nitrogen sources (soytone and yeast extract being minor C and N sources, respectively, Materials and Methods), *Lp* acidifies the environment by secreting organic acids, and *Ca* alkalizes the media by cleaving urea into ammonia (Fig. 1B). Given that *Lp* and *Ca* prefer acidic and alkaline environments, respectively, the two species produce environmental pH values that they can tolerate, but the other species cannot. Accordingly, both species cannot coexist, and one outcompetes the other, where the initial relative abundance determines the winner [16].

We tested whether a different past could change the fate of our two-species model system (Fig. 1C and described in more detail in the Materials and Methods). *Lp* and *Ca* were grown independently, free to change their environment. Subsequently, cells of the interaction partner were added without their environment (i.e. after washing them) to obtain communities with similar species abundances (Fig. 1B). Despite having similar compositions, the communities differ in how they reached these initial compositions and, thus, their pasts. Only *Lp* or *Ca* could keep their established environmental changes, whereas for the interaction partner, this altered environment was removed. The experiment was performed for different fractions of the two interaction partners (see Materials and Methods).

After mixing the interaction partners, we cultivated them under identical conditions with daily dilutions into fresh media over four days. The final community composition was analyzed by counting colony-forming units (CFU, see Methods). As an overall trend, we can see that the competition outcome depends on the initial mixing ratio of the two species (Fig. 1D). The higher the initial abundance of a species, the more likely it wins the competition, i.e. at a high initial abundance of *Lp*, *Lp* tends to win, and at a high initial abundance of *Ca*, *Ca* tends to win—the system is bistable. However, the interaction outcome is not only determined by the relative abundances of the two species. In the range from around 4% to 46% initial abundance of *Ca*, the system shows different outcomes depending on its history, even for the same initial relative abundances. The memory effect, therefore, impacts interaction outcomes over a rather large range of mixing ratios, which would correspond to different dispersal rates in a natural invasion process. The species that previously modified the environment had a higher chance of winning, thus exhibiting that the system's past impacts the interaction outcome. The change of the environment is both sufficient (Supplementary Fig. 2) and necessary (Supplementary Fig. 1, Supplementary Figs 3 and 6) to cause the observed memory effect, which therefore does not seem to depend on intracellular memory.

To investigate whether the collective change of the environment causes the externalized memory effect, we repeated the experiment at lower cell densities. If collective actions of the communities are necessary for memory formation, it should be weakened or disappear at low cell densities, which we indeed observed (Fig. 1E) The difference between the two conditions (blue and red lines in Fig. 1D) disappears upon diluting the co-cultures (Fig. 1E and Supplementary Fig. 1). In a similar way, the memory effect is also reduced at lower nutrient concentrations that allow for less microbial growth (Supplementary Fig. 3). Thus, we can show here that bacteria can build up an externalized memory as a consequence of collective action that impacts future interaction outcomes.

Community assembly can lead to different outcomes even for the same initial composition in the presence of externalized memory (Fig. 1). Accordingly, the history of a microbial community can decide its future composition. These observations raise the question of whether a similar mechanism can cause microbial communities to develop differently based on past events like different growth conditions. The metabolic state of bacteria changes with environmental conditions such as temperature [17, 24, 25], oxygen concentration, and the availability of resources. Accordingly, how bacteria change the environment depends on these growth conditions, and different past growth conditions may impact future interaction outcomes differently. To test this idea, we grew our two model strains, *Lp* and *Ca*, under different conditions each. The temperature was either kept at 18°C or 35°C, with the latter being preferred by both strains (Supplementary Fig. 4A). The oxygen levels were either kept low (preferred by *Lp*) [26] or at atmospheric conditions (preferred by *Ca,*  Supplementary Fig. 4) as described in the Materials and Methods. *Lp* grown under preferred conditions was mixed with *Ca* grown under unpreferred conditions and vice versa (Fig. 2A)*.* In this way, similar community compositions were obtained, but from bacteria grown under different conditions (Fig. 2A). After mixing, the communities were all grown under daily dilution for four days. Analysis of final community compositions demonstrate that different past growth conditions result in different interaction outcomes (Fig. 2B). Again, this memory effect disappears upon dilution, i.e. at low cell densities (Supplementary Fig. 5), as well as the result of removing externalized memory (Supplementary Fig. 6). To understand better how the different growth conditions could impact the interaction outcomes, we measured how bacteria change the pH—the major driving factor of bacterial interactions in this system [16]. Empirical evidence indicates that the pH change per colony-forming unit is higher in preferred vs. unpreferred conditions for both species (Fig. 2C; the impact of oxygen levels and temperature alone are shown in Supplementary Fig. 4). These results suggest that past growth conditions—in this case, temperature and oxygen—can lead to different environmental changes by the bacteria and, thus, different future interaction outcomes.

### Mathematical model shows externalized memory

Our experiments showed that collective memory influences bacterial interactions over time. To get a better mechanistic understanding of these results, we formulated a mathematical model based on differential equations. In this model, species interact only through the environment, that is, they change an environmental variable *p,* where the variable’s value determines the bacteria’s growth (Fig. 3A). The bacteria can only grow if this environmental variable lies within a specific range (corresponding differential equations provided in Fig. 3A). *N_i_* is the population density of species *i*, *p* is an environmental variable (e.g. pH, toxins or oxygen concentration) that can be changed by the bacteria and *p_o,i_* is the optimal value of *p* for species *i*. θ is the Heaviside function that becomes equal to one only if its argument is positive, that means if *p* is close enough to *p_o,i_*. *r_dilution_* corresponds to a dilution factor that removes cells from the system and, therefore, equals the death rate of the bacteria. At the same rate *r_dilution_*, fresh media with *p* = 0 (negative *p* is allowed in this model) is added to the system. The environmental parameter *p* is changed by the species *i* with the rate *k_i_*. We used a similar framework in the past to describe environment-mediated interactions in bacteria [22].

To recapitulate our experimental findings that collective memory can impact interaction outcomes theoretically, we simulated the interaction of two species that lower or increase the environmental parameter *p* and prefer low or high values of *p* (*p_o,i_*), respectively. The two species, therefore, change the environment in a way that is beneficial for themselves but detrimental for the interaction partner. We first grew one of the species and mixed it with cells of the other species as done experimentally before (Fig. 1, Materials and Methods). Indeed, we observed a similar memory effect in the model that also disappears upon diluting the system (Fig. 3B). It is important to highlight that such a memory effect where for the same initial species abundances, different interaction outcomes are obtained is only possible because the interaction is mediated through the environment. The environment acts as a third variable (Supplementary Text). Moreover, the memory effect only occurs if the change of the environment by the species (*k_i_*) is not too fast (Fig. 3C). If the bacteria change the environment too rapidly, the memory is “overwritten” by the bacteria and thus cannot influence the interaction outcomes (see also Supplementary text). Importantly, the system must be multistable to stay over long times in different states and show the observed memory effect. A monostable system will return to its only stable state over time [27].

To theoretically explore the possible impact of collective memory on more complex communities, we also simulated the assembly of multispecies systems in the presence of collective memory. We performed the simulations for eight species systems and three environmental variables (Materials and Methods and Supplementary Text 1). One species was grown, and then, cells of the other species were added to the system to obtain the same initial abundances but with different histories (Fig. 3D). This was repeated for different abundances of the same set of species (Supplementary Fig. 7A). The dynamics of the system were simulated to obtain the final composition at the end of the simulation run (Supplementary Figs 8 and 9). These final states are visualized after reducing the 8D outcome (one dimension for each species) to a 2D plot by applying NMDS to the Bray–Curtis dissimilarity matrix of the obtained final communities (Fig. 3C, Materials and Methods). We notice that our theoretical model system shows multistability even without memory. When the cells are mixed in different ratios in fresh media, the system ends up in one out of four final states (Fig. 3E red and Supplementary Fig. 7F). The frequency for the different initial community mixtures to end up in one of the states was quantified (Fig. 3F, left). In the presence of externalized memory, we observe two interesting phenomena. First, the relative frequency of ending up in one of the four alternative states changes depending on which species’ memory the assembly takes place in. Second, the systems can end up in novel states that could not be reached in the absence of memory (yellow and orange bars in Fig. 3F). Replacing the Heaviside function in Equation (1) (Fig. 3A) by a Gaussian function leads to similar outcomes (Supplementary Fig. 10). Similar findings were observed in simulations with a second set of bacteria (Supplementary Fig. 11). Overall, these simulations suggest that the assembly of the same set of species with the same initial abundances can nevertheless end up in different communities in the presence of externalized memory.

### Externalized memory can impact community assembly

**Figure 4:**
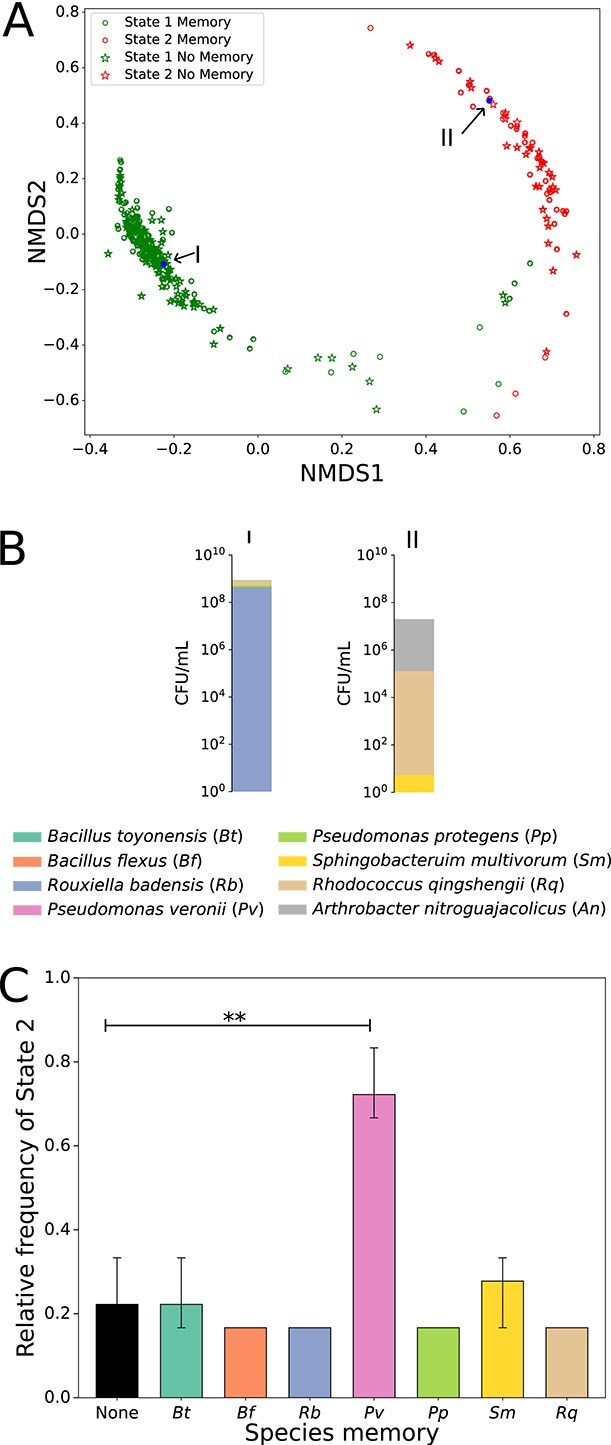
Collective memory impacts community assembly. (A) Eight-species communities were mixed with varying initial compositions either in the absence or presence of different memories (three technical replicates for each condition, see Supplementary Fig. 12). NMDS plots based on Bray–Curtis dissimilarities between final community compositions depict the communities ending up in two final states, referred to as State 1 and State 2. The state for each community is obtained by hierarchical clustering performed on Bray–Curtis dissimilarities (Materials and Methods). (B) State 1 is dominated by *Rouxiella badensis* (*Rb*) with relatively high total cell densities, and State 2 is dominated by R. *o*ingshengii (*Rq*), S. *m*ultivorum (*Sm*), and A. *n*itroguajacolicus (*An*) with relatively lower total cell densities. (C) Exposure to the externalized memory of P. *v*eronii during the initial community assembly significantly increases the frequency of community ending up in State 2 as compared to absence of memory (Mann–Whitney *U* test, *Pv* vs none, *P-*value = .003). Lines on bars represent 95% confidence intervals calculated by bootstrapping within each community of each species’ memory.

We found theoretically that collective memory can also influence the assembly of more complex communities. To test this result experimentally and to study further how common this phenomenon is in an arbitrary set of bacteria, we tested the impact of collective memory on community assembly experimentally (Fig. 3). We randomly selected eight bacterial species from our C. elegans gut strain collection for community assembly. To estimate the impact of externalized memory on the assembly process, the eight species were mixed in different abundances, where the memory of only a single species was present in the system during assembly (equivalent to Fig. 3D). This corresponds to a situation where one species grows first in its habitat and changes the chemical composition, followed by the arrival of the other bacteria. We tested six initial community compositions (Supplementary Fig. 12A), with eight different memories in three replicates each. This results in 144 community assembly processes, along with replicates assembled in the absence of memory. After mixing, we cultivated the communities for 12 days under daily dilution into fresh media, i.e. all communities were treated the same after the initial setup. The compositions of the communities were assayed on Days 5, 10, and 12 of the experiment by plating on NM agar and counting the forming colonies (Materials and Methods, Supplementary Figs 13–16, Supplementary Fig. 18). Bray–Curtis dissimilarities between the communities of Day 12 were calculated, followed by dimensionality reduction using NMDS (Fig. 4A).

Our experiments of community assembly in the presence of collective memory show that the different communities converge toward two final states, i.e. the system is multistable. The two states are differentially colored based on the hierarchical clustering of the final communities’ Bray–Curtis dissimilarities (Fig. 4A, Supplementary Figs 12C and 17). This observation also aligns with the visual impression of the raw data (Supplementary Fig. 16). Multistability seems to be a relatively common feature of microbial communities, as we will discuss in a separate publication. The different initial communities collapse either into State 1, which is dominated by *Rouxiella badensis* (*Rb*), or State 2, which is dominated by Arthrobacter nitroguajacolicus (*An*)*,* Rhodococcus qingshengii (*Rq*)*,* and Sphingobacterium multivorum (*Sm*) (Fig. 4B). In the absence of memory, the communities end up mostly in State 1 and only around 22% of the cases in State 2 (Fig. 4A and C, left). The states in which the communities end up are mainly determined by the initial species composition (Supplementary Fig. 12E). In the presence of collective memory, the community assembly outcomes change (Fig. 4C, Supplementary Fig. 12D). In particular, the memory of P. veronii significantly alters the community assembly (ANOSIM [28], calculated on Bray–Curtis dissimilarities for P. veronii vs no memory, *P*-value = .001, *R* value = 0.426) and significantly increases the frequency of communities ending up in State 2 (Mann–Whitney *U* test, *Pv* vs “no memory”, *P-*value = .003). In contrast, communities assembled under other memories were not significantly different from those assembled without memory. Overall, the externalized memory of specific strains can strongly impact community assembly, whereas at the same time, different communities can also be variably susceptible to this memory effect (Supplementary Fig. 16). We cannot fully exclude that internal memory impacts the assembly of this eight-species community, but our data show that externalized memory can alter the outcome of the assembly process. Although the memory of P. veronii impacts the community assembly, P. veronii*’*s memory does not ensure its survival in the final community (Supplementary Fig. 16).

## Discussion

Despite their simplicity, bacteria can store memory in many ways [29, 30], allowing them to retain cellular states over long periods of time [31–34]. Bacterial growth and gene expression can depend on their past growth conditions [35–38], and the virulence of pathogenic bacteria can be lastingly altered by a previous host contact [39]. Bacteria can store these memories inside the cell, e.g. by retaining active proteins in the cytosol over longer times or showing persistent gene activation even after the stimulus disappeared [36, 40, 41]. In many cases, however, the mechanistic origin of memory is not understood [32, 35, 39] and may not even be adaptive in nature but just side effects of complex biological systems [5].

Our work describes a different type of bacterial memory that is not stored inside the cell but externalized in the environment. Bacteria can generate external memory by collectively changing the chemical composition of the environment [15, 16, 42, 43], which is often called niche construction or ecosystem engineering in the ecological context. These environmental changes may depend on events in the past that, in this way, impact interaction outcomes in the future. Because single bacteria can usually not significantly change the environment, whole populations must work together to form this external memory. Indeed, bacteria are well known for collective traits [44], like cooperation [45] or quorum sensing [46], which aligns with their community lifestyle [47].

Collective memory has been theoretically described for animal groups such as fish swarms. In such animal groups, specific swarming patterns may appear and persist over time only due to the interactions among the groups’ individuals but without the memory stored inside the individuals [48]. Another example of externalized, collective memory in nature is pheromone trails, which occur in social insects [49]. Ants can deploy a pheromone trail back to their nest upon finding food to guide other ants, although these ants do not have an intrinsic memory of the food source. The volatility of pheromones sets the period of this collective memory; the faster they evaporate, the shorter the memory is [49]. Pheromones are particular substances that impact behavior. However, we show that even rather unspecific by-products of metabolism that did not evolve for memory storage can function as ingredients of an externalized memory in bacteria, as was investigated theoretically [50].

The collective memory that we describe here connects to several concepts of memory in ecosystems, and an overview of memory effects in ecological systems is provided (Supplementary Table 1). Thus, the ability of a new invader to establish itself in a community may not just depend on the community’s composition but also on its past. In that regard, the memory effects we described here can be an underlying mechanism causing what is known as the priority effect [51–53]. In the priority effect, the order of arriving species determines the assembly of communities. Chemical modifications of the environment by a microbial population or ecosystem could, therefore, over longer times determine which species can invade the ecosystem and which cannot. Indeed, we recently found that modification to the growth media can lead to complex and emergent invasion patterns, and even completely protect microbial populations against invasions [54]. Accordingly, externalized memory may influence how resistant host-associated microbiota are to invading pathogens. In soil environments, bacteria are thought to inhabit small water droplets [55], which enable them to locally modify their surroundings. These localized environmental changes may give rise to memory effects that influence local resistance to invasion, thereby shaping microbial biodiversity.

Ecosystems are always the outcome of their historical development; for example, in macroscopic ecosystems, past rainfalls [6] or land usage [7] can impact future ecosystem development. The presence or absence of species in an ecosystem also depends on whether these species historically invaded this ecosystem or died out at some point [56]. Lasting modifications of the environment (ecosystem engineering) produced by an ecosystem’s organisms could shape trajectories even in macroscopic ecosystems. However, outside our very controlled lab experiments, it may often be difficult to decipher the underlying mechanisms.

Bacteria that change the chemical composition of their environment construct an ecological niche [8, 15, 16, 57–59]. We show here that different niches could be constructed depending on the past conditions, and differently constructed niches accordingly impact the future development of ecosystems differently. Similar statements have been made about human niche construction, as in the case of agriculture, where human modifications of the environment can be a response to changing environmental conditions (e.g. climate change), and anthropogenically altered environments can impact humans and nature over a long time [60, 61]. However, the memory effects we describe in this work are the combined outcome of environmental changes and a multistable system, where different changes push the multistable system into different final states. Because of this multistability, the system stays in the reached states even after the modification of the environment is gone, causing the observed long-term memory effects. A monostable system returns to the same state when the environmental change (i.e. constructed niche) vanishes.

On the theoretical side, externalized memory may have overlooked consequences. Often, Lotka–Volterra models are used to model microbial communities for theoretical insights [62] or to predict experimental outcomes [63–65]. However, these models only account for direct species interaction—i.e. not mediated through the environment—and thus cannot describe externalized memory. Indeed, Lotka–Volterra models can strongly deviate from models that consider environmental changes because they ignore lasting environmental changes [ 66, 67].

On the eco-evolutionary scale, externalized memory may be able to ensure the long-term survival of bacteria within an ecosystem by biasing future interactions in their favor. However, in how far externalized memory is the outcome of evolutionary adaptation or just the effect of metabolic by-products remains currently unclear. The microbial communities in our guts are connected to our health in many ways [68, 69], and accordingly, long-lasting changes within this microbiota could impact our well-being [70]. Indeed, the gut microbiota has been shown to possess a memory of past nutrient exposure, medical treatments, or infections [71–73]. Still, given the intrinsic complexity of these systems, it is very challenging to identify the underlying mechanisms. At least in a simple model system of a bacterial gut community, memory was suggested to be caused by the interplay between bacterial metabolism and modification of the environment [74]. Externalized memory may, therefore, also play a role in host-associated microbial communities and be of medical relevance. In particular, medical treatments or dietary changes [75, 76] can alter the pH in the gut microbiota, which can cause externalized memory, as we have shown here.

## Supplementary Material

Supplementary_text_wraf173

## Data Availability

The datasets generated and/or analyzed during the current study are available in the Dryad repository (DOI: https://doi.org/10.5061/dryad.ksn02v7hp).

## Code availability

Python code of the simulations and parameters to reproduce shown figures can be found at https://github.com/RatzkeLab.
