# Supplementary material for: Environment-mediated interactions cause an externalized and collective memory in bacteria
Source: ISME J. 2025 Aug 11;19(1):wraf173. doi: 10.1093/ismejo/wraf173 (PMC12456175; doi:10.1093/ismejo/wraf173)
Supplement: Supplementary_text_wraf173 [file supplementary_text_wraf173.pdf]

Supplement: Environment-mediated interactions cause an externalized and collective memory in microbes

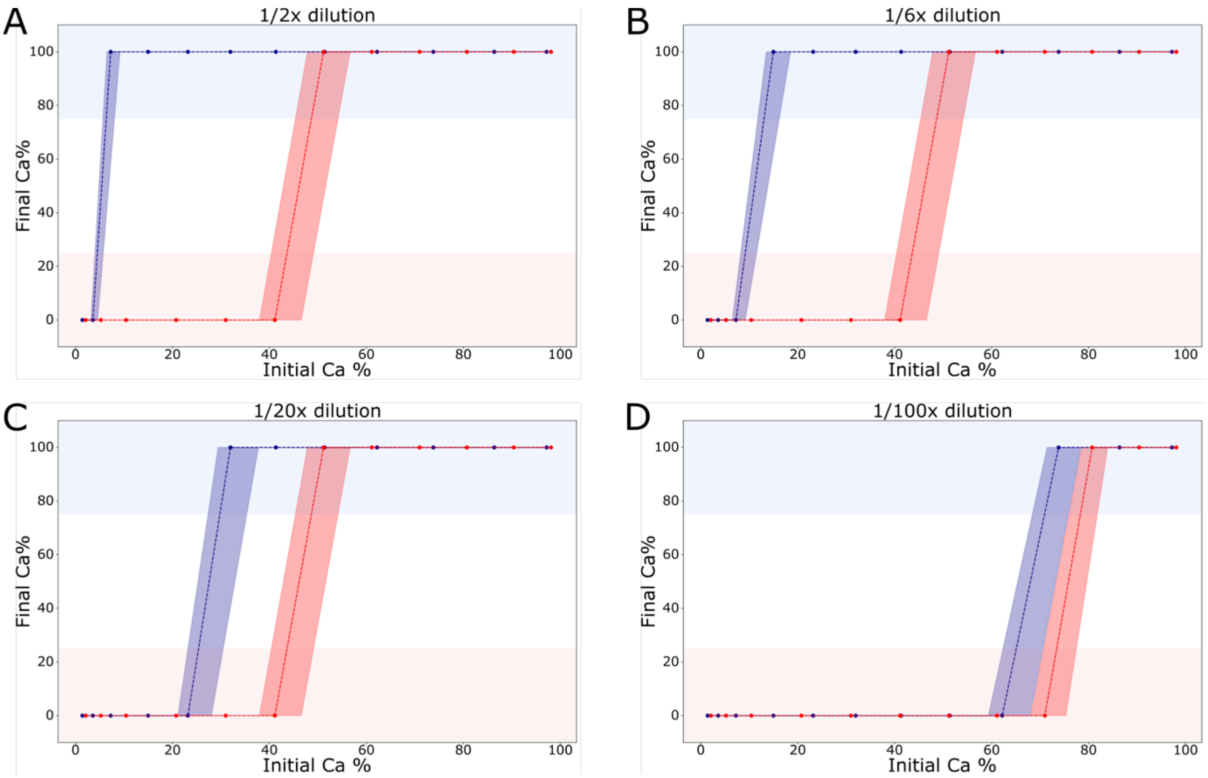

Supplementary Fig. 1: Lowering the population density decreases the memory effect. The microbial co-cultures are diluted with fresh media after mixing with different ratios, leading to successive loss of the memory effect with increasing dilution ratios. **(A)** 1/2x dilution (n = 3) (referred to as Diluted co-culture (1/2x) in Fig. 1D). **(B)** 1/6x dilution, **(C)** 1/20x dilution (n = 3) and **(D)** 1/100x dilution (n = 3) (referred to as Diluted co-culture (1/100x) in Fig 1E) where the memory effect completely disappears. Diluting the co-culture reduces both the population densities of the present species as well as the collective memory they produced. Consider that **(A)** to **(B)** dilutions as well as **(B)** to **(C)** are by a factor of roughly 3, whereas **(C)** to **(D)** dilution is by a factor of 5. Also, because x-axis is binned, we do not observe small shifts in change in curve in *Lp* memory curves in **(B)** and **(C),** but it is evident in **(D).**

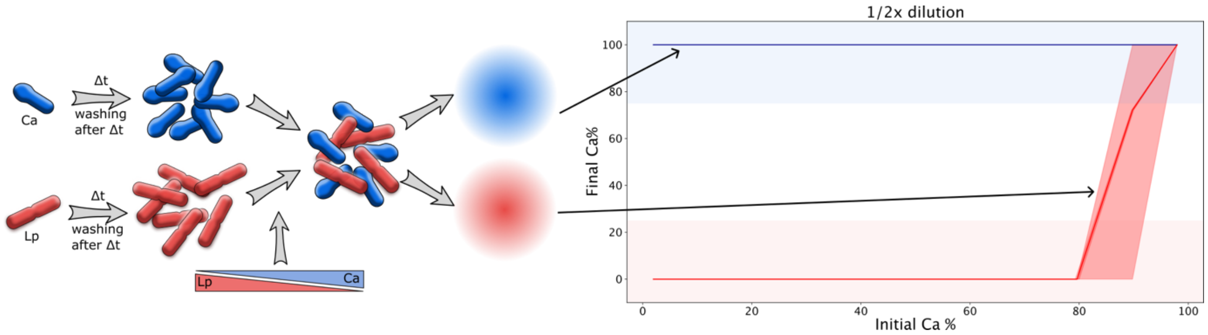

Supplementary Fig. 2: Modified media is sufficient for the externalized memory effect. To show that the different interaction outcomes shown in Fig. 1 are indeed caused by an externalized memory (i.e., modification to the growth media) instead of an internal memory (cellular memory), we recapitulated experiment in Fig. 1 with a different approach. Memories were removed from microbes by centrifuging cells, taking off supernatants, and washing the cells. The filtered supernatants were then added back to the bacterial co-cultures after mixing both bacteria followed by 1/2x dilution into fresh media and daily dilution into fresh media for 4 days (n = 3). As can be seen in the figure above, adding different supernatants to the same mixture of cells is sufficient to observe different outcomes.

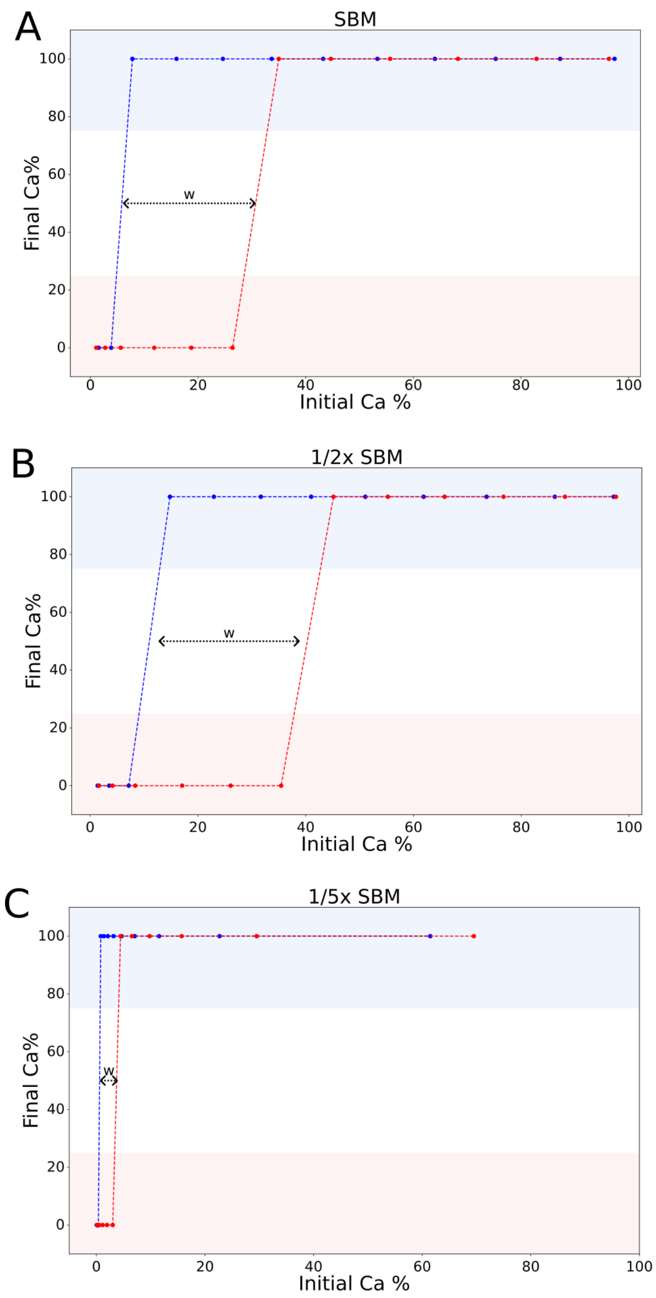

Supplementary Figure 3: Limiting the nutrients during the formation of 'externalized memory' leads to a decline in the memory effect. Lower nutrient concentrations lead to reduced growth of the microbes and should therefore also lower the environmental change caused by the bacteria. Accordingly, the memory effect should be decreased, which can indeed be observed by diluting the media from 1x **(A)** over 1/2x **(B)** to 1/5x **(C)** (n=1 for each). The windows size (w) which is the difference between the two curves at final *Ca*% equaling 50% accordingly decreases from **(A)** w=28% to **(B)** w=29% and **(C)** w=3%.

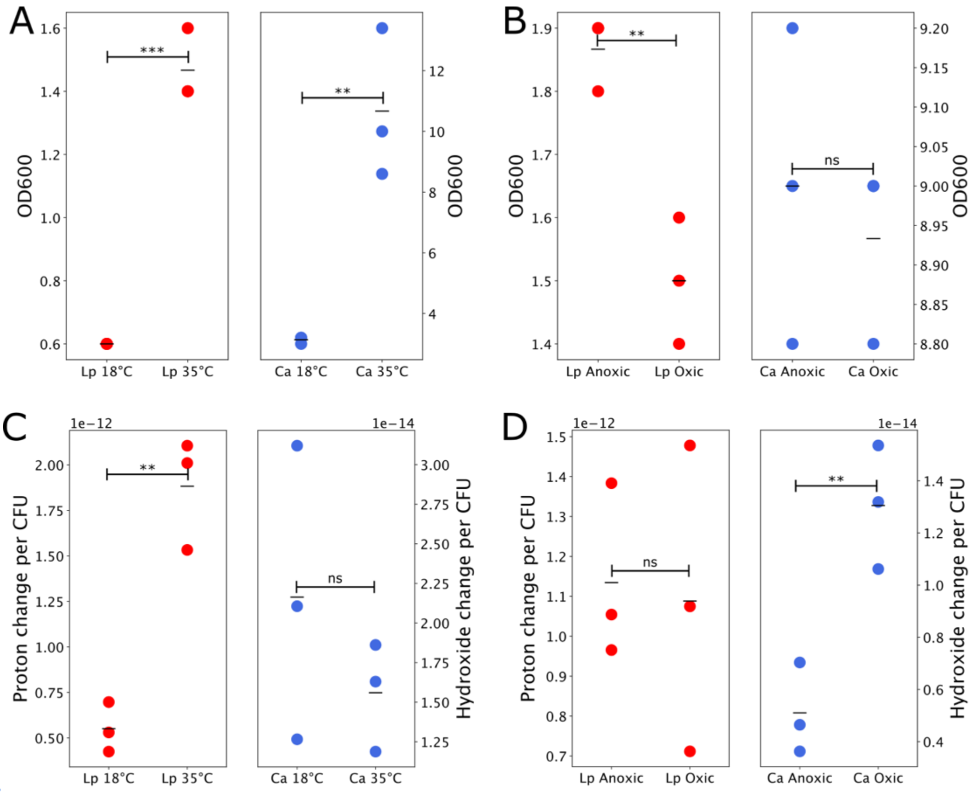

Supplementary Figure 4: Effect of single growth conditions (Temperature or Aeration conditions) on growth and proton change per CFU of *Lp* and *Ca* (A) Both *Lp* and *Ca* grow significantly better at preferred temperature (35°C) as compared to growth at 18°C (n = 3, two-tailed Student's t-test, left *P* value = 0.000202. right *P* value = 0.00616). **(B)** *Lp* grows significantly better in anoxic condition as compared to oxic conditions (n = 3, two-tailed Student's t-test, *P* value= 0.005328), whereas *Ca* growth in oxic or anoxic conditions is not significantly different. **(C)** We observed significantly higher proton change per CFU when *Lp* is grown in preferred temperature (n = 3, two-tailed Student's t-test, *P* value = 0.0023599), whereas in case of *Ca*, changing growth temperature did not have significant difference on Proton change per CFU (n = 3, two-tailed Student's t-test, *P* value= 0.3499) **(D)** On the contrary, changing growth aeration conditions does not significantly change proton change per CFU for *Lp* (n = 3, two-tailed Student's t-test, *P* value = 0.86501), whereas in case of *Ca,* proton change per CFU is higher in Oxic conditions as compared to growth in anoxic conditions (n = 3, two-tailed Student's t-test, *P* value = 0.0094949).

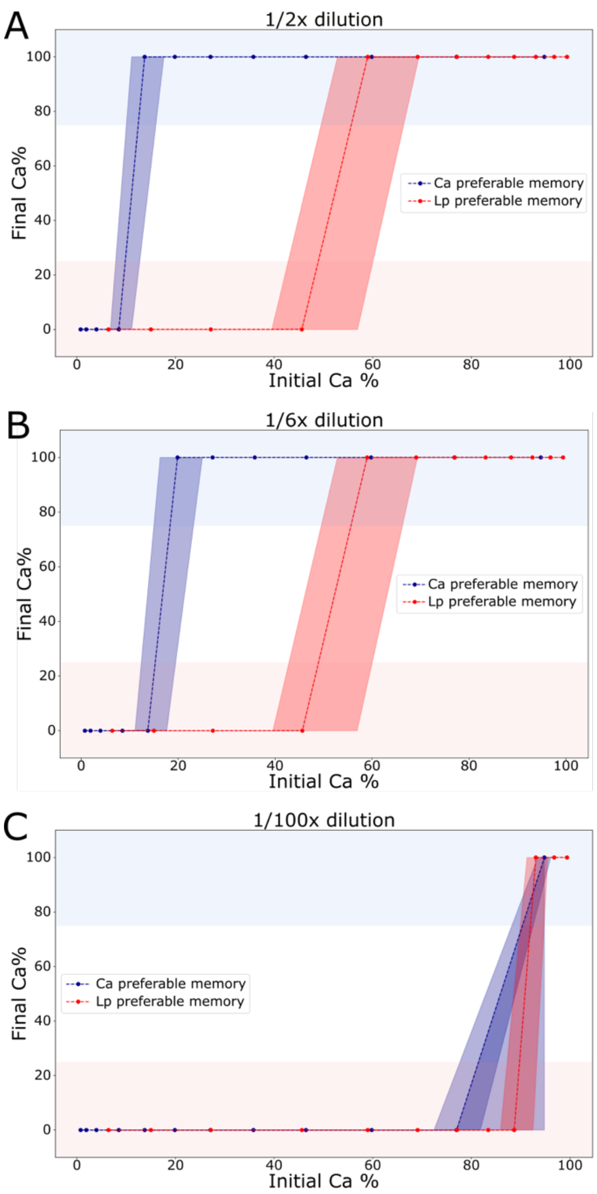

Supplementary Fig. 5: Also externalized memory caused by different growth conditions disappears as co-cultures are diluted. We performed the same measurements (as in Fig. 2) but diluted the co-cultures after mixing by different ratios (analog to what we show in Supplementary Fig.1 for the experiments depicted in Fig. 1, n=3). Again, the memory effect successively gets weaker with increasing dilution rate from **(A)** 1/2x dilution (Diluted co-culture (1/2x) in Fig. 2B) to **(B)** 1/6x dilution and disappears at **(C)** 1/100x dilution. Consider that **(A)** to **(B)** dilution is by a factor of 3, whereas **(B)** to **(C)** dilution is by a factor of roughly 16. Also, because x-axis is binned, we do not observe small shifts in the *Lp* memory curves in **(A)** and **(B),** but it is evident in **(C).**

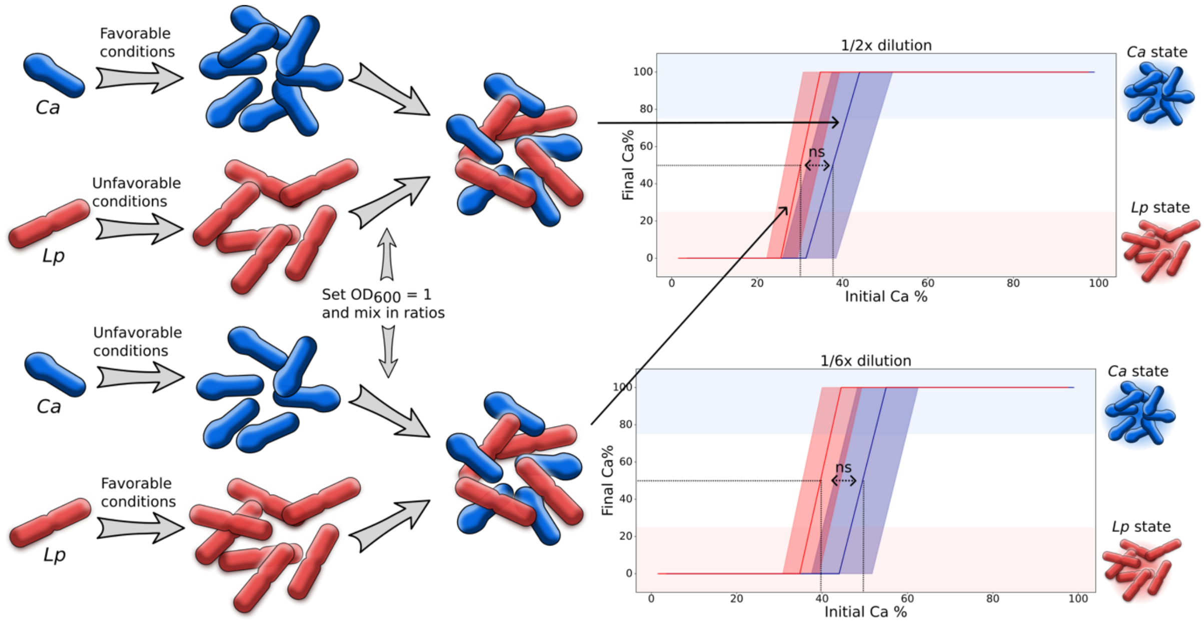

Supplementary Fig 6. Removing spent media removes the memory effect. Aimed at verifying the role of 'supernatant' in the memory effect, we established an alternative 'depletion' approach, where memories were not re-added to 2-member communities assembled from microbes grown in favorable and unfavorable conditions (Fig. 2). The results demonstrate that there is no internal memory in the microbes that impacts the interaction outcomes. Lack of significance in the plots is determined by two-tailed Student's t-test based on values of Initial *Ca* percentage (x-axis values) corresponding to final *Ca* percentage = 50% for each curve (n = 2).

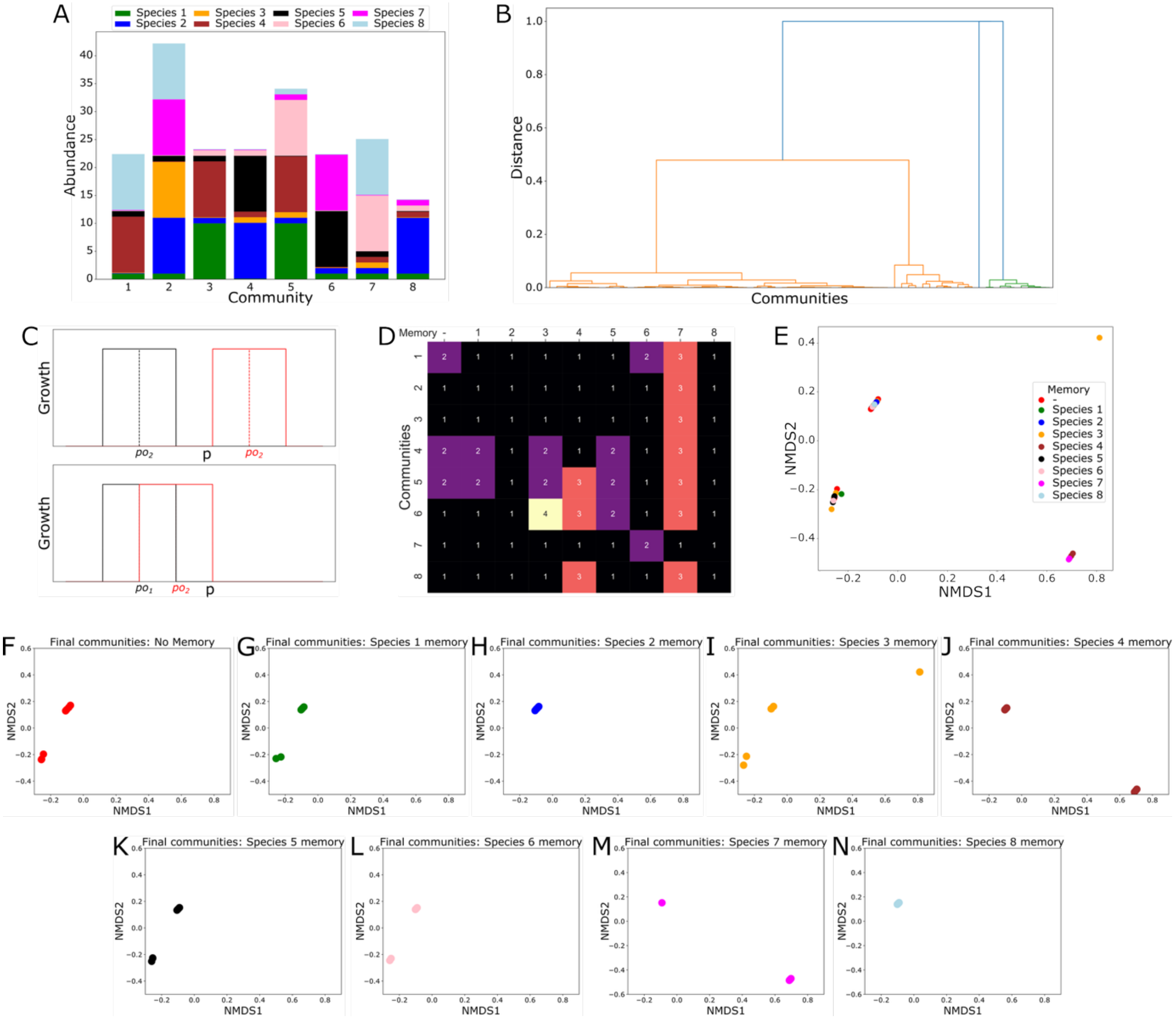

Supplementary Fig 7: Simulation of the first set of species using equations 1 and 2. Plots in Fig. 3 as based on these results. **(A)** Initial composition of initial communities. Each community is composed of 8 species, where each abundance is randomly selected from set [0.1,1,10]. **(B)** Hierarchical clustering of final community compositions across all initial compositions and supernatants reveals 4 clusters (also referred to as states). **(C)** In order to ensure growth of both microbes, optimum P values of species within the community must be close to each other, such that their 'growth zones' coincide. **(D)** Heatmaps depicting states reached in the presence of different memories across all communities. State 3 is primarily achieved in the presence of Species 7 memory (sometimes also in species 4 memory), whilst communities can end up in either state 1 or 2 in absence of memory. **(E)** Similarly, NMDS plot based on Bray-curtis dissimilarities between final community compositions across all communities shows clustering into 4 distinct clusters, (similarly observed in Hierarchical

clustering). **(F), (G), (H), (I), (J), (K), (L), (M), (N)** Single species memory (or absence thereof) NMDS plots from (E).

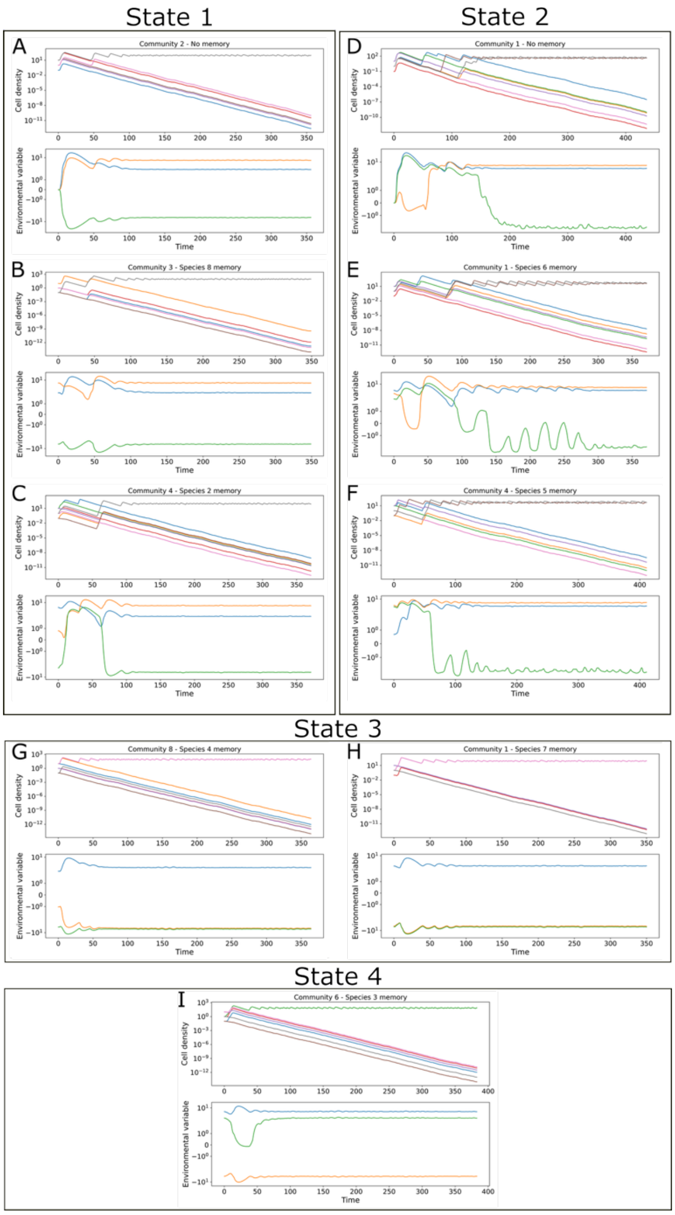

Supplementary Fig 8: Example plots from simulations of equations 1 and 2, depicting cell density and environmental variables over time. Bray-Curtis dissimilarities were calculated from the final community compositions, which in turn were used for non-metric multidimensional scaling as well as hierarchical clustering (Fig. 3). Clustering revealed 4 clusters, which are hereby referred to as states. Several examples of interesting dynamical behaviors are shown. Different communities differ in their initial abundances and in absence of any memory may end up in different states, as

observed in **(A), (D)**. Identical communities can end up in different states in the presence of different memories initially, as seen in **(C), (F)** and in **(E), (H)**.

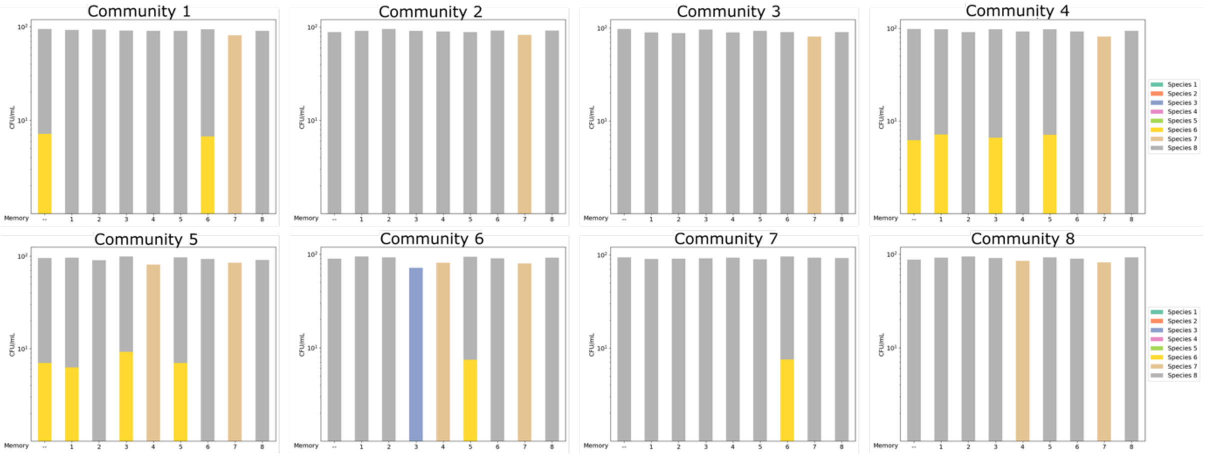

Supplementary Fig 9: End-point relative abundances of 8 different communities, derived from simulations based on equations 1 and 2. Communities were built from the same eight species but with different relative abundances (see Supplementary Fig. 9), and their growth was simulated in the presence of different memories or the absence of memory. Each plot (community) represents sample communities that began with identical relative abundances but varied in their memories. The outcomes are plotted in simplified form also in Fig. 3 and Supplementary Fig. 9.

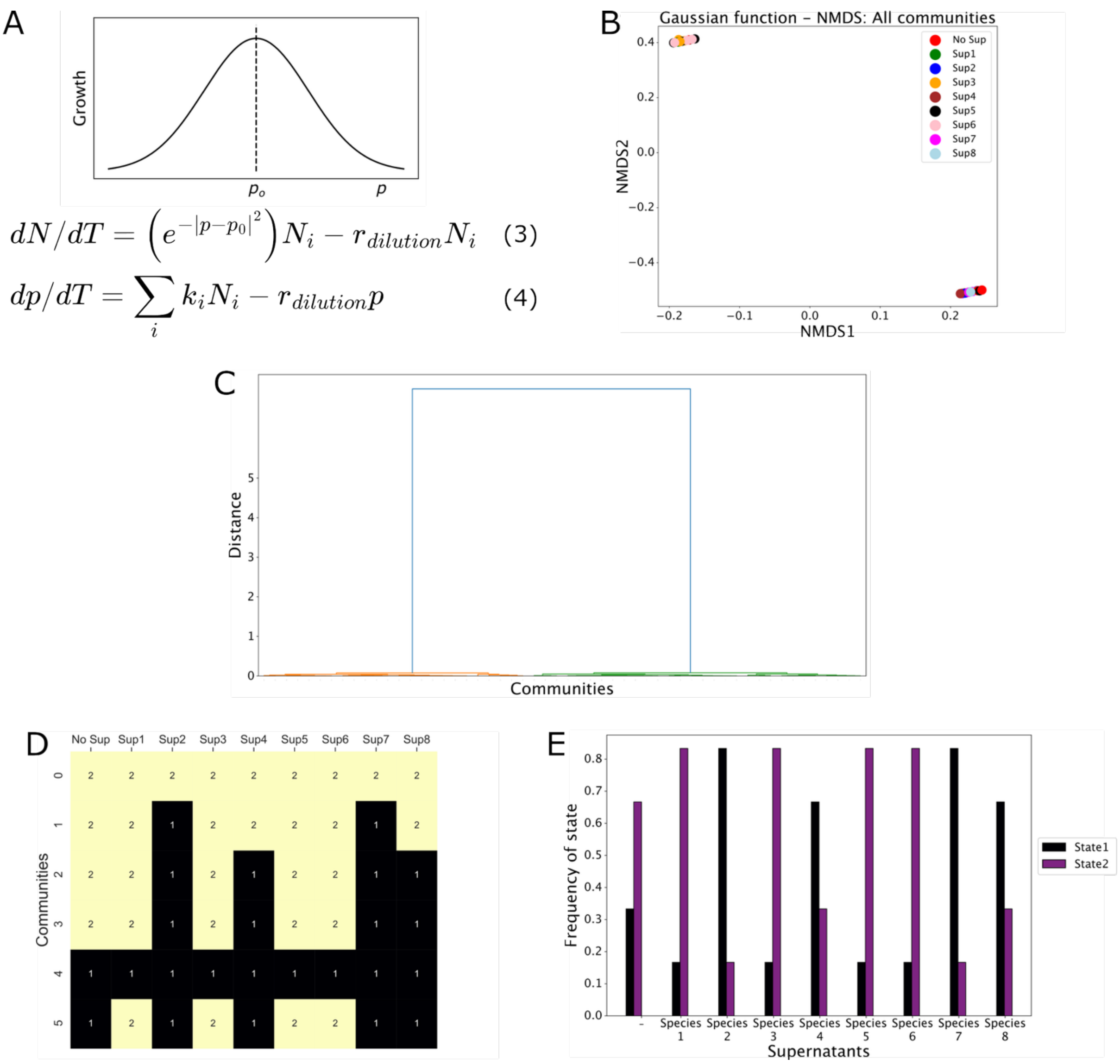

Supplementary Figure 10: Replacing the Heaviside function by Gaussian function in community simulations leads to similar results. (A) We tested a modified model in which the Heaviside function was replaced by a Gaussian function with the highest growth obtained at p_o. **(B)** 6 communities (with randomly generated community composition from a subset of [0.1,1,2,10] for each species density) were simulated using equations 3 and 4. Bray-Curtis dissimilarities were calculated from final community compositions and followed by dimensionality reduction (NMDS). The obtained NMDS plot shows 2 distinct clusters, which is backed up by **(C)** Hierarchical clustering based on Bray-Curtis dissimilarities identified two clusters (state 1 and 2) **(D)** Heatmap of communities' final states for each initial community and memory **(E)** Higher frequency of State 1 is observed in communities simulated in different memories (Species 2, 4, 7, 8) as compared

to communities simulated without memory. Such results prove analogous to what we observe with simulations performed using the Heaviside function (Figure 3).

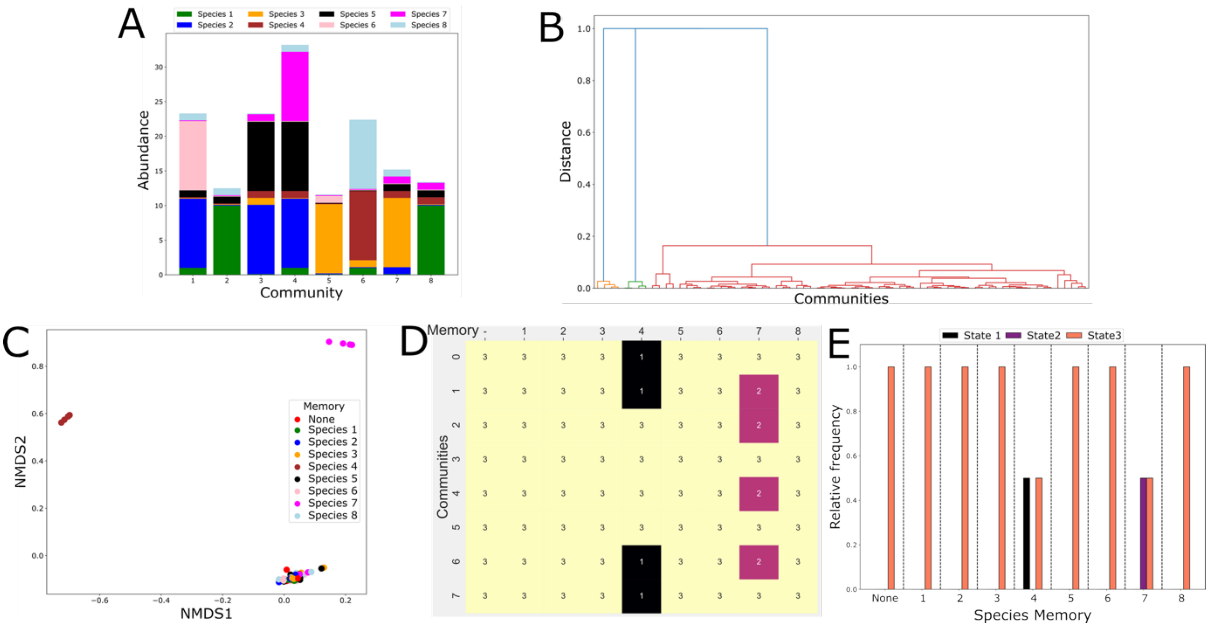

Supplementary Fig 11: A second set of simulated species shows memory dependent outcomes as well. To get an impression of how general memory effects are in our modelling framework, the same simulations (as in Supplementary Fig. 10) were repeated with a second set of "species". **(A)** The initial composition of 8 communities using a second set of species. Each community is composed of 8 species, where each species' abundance is randomly selected from the set of [0.1,1,10]. **(B)** Post simulation, Bray-Curtis dissimilarities were computed for the final community compositions. Hierarchical clustering based on these dissimilarities reveals 3 major clusters (referred to as states). **(C)** Similarly, NMDS projection of Bray-Curtis dissimilarities between final communities shows convergence of communities into three clusters. **(D)** Heatmap of states achieved in different communities in the presence of memories from individual constituent species. As observed also in Supplementary Fig. 9, some states are only observed in the presence of memories of specific species, which suggests that some states of microbial communities may only be reached in the presence of the right memory. **(E)** Presence of memories from either species 4 or species 7 leads to an increase in frequency of community ending up in either state 1 or 2 respectively.

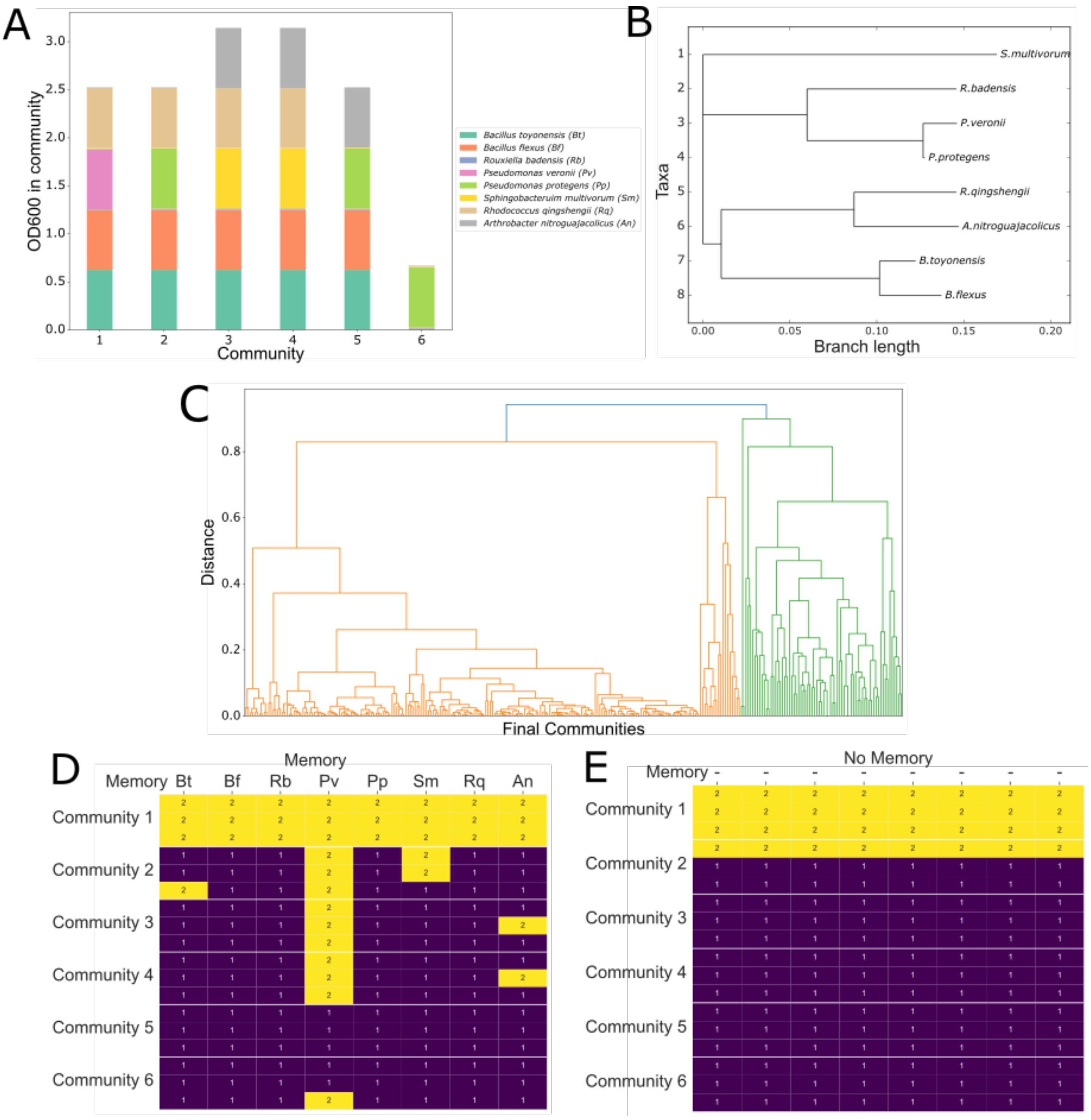

Supplementary Fig 12: Assembly of 8-species communities is affected by externalized memory. Initially, 6 different communities were assembled from the same 8 species, but with different initial abundances. The communities were incubated with 12 daily dilutions into fresh media. On the first day either memory of other species (n=3 for each memory, (D)) or no memory (n=24, (E)) was present (see Methods for details). Initial absolute abundances as OD600 of each species are shown in **(A)** Equi volume proportions of each species at either high (OD600 = 5) or low (OD600 = 0.05) ODs were mixed into 6 different communities. (**B)** Bacterial species used in the community assembly represent diverse taxa as shown by a phylogenetic tree. (**C)** The final community composition was obtained by plating after 12 days of daily dilution and counting each species' number of forming colonies (CFU, see also Supplementary Fig. 12, 16). Bray-Curtis dissimilarities were computed. Hierarchical clustering based on these dissimilarities revealed 2 major

clusters. The cluster identities of all assembled communities were used to generate heatmaps shown in **(D)** in the absence of another species' memory and **(E)** in the presence of memory. The x-axis represents memories of different species and on y-axis different communities. Horizontal white lines separate 3 technical replicates of each community. In particular, the memory of *Pseudomonas veronii* leads to communities ending up in State 2 more frequently as compared to memories of other species as well as communities assembled in absence of supernatant. For technical details the main text and Methods.

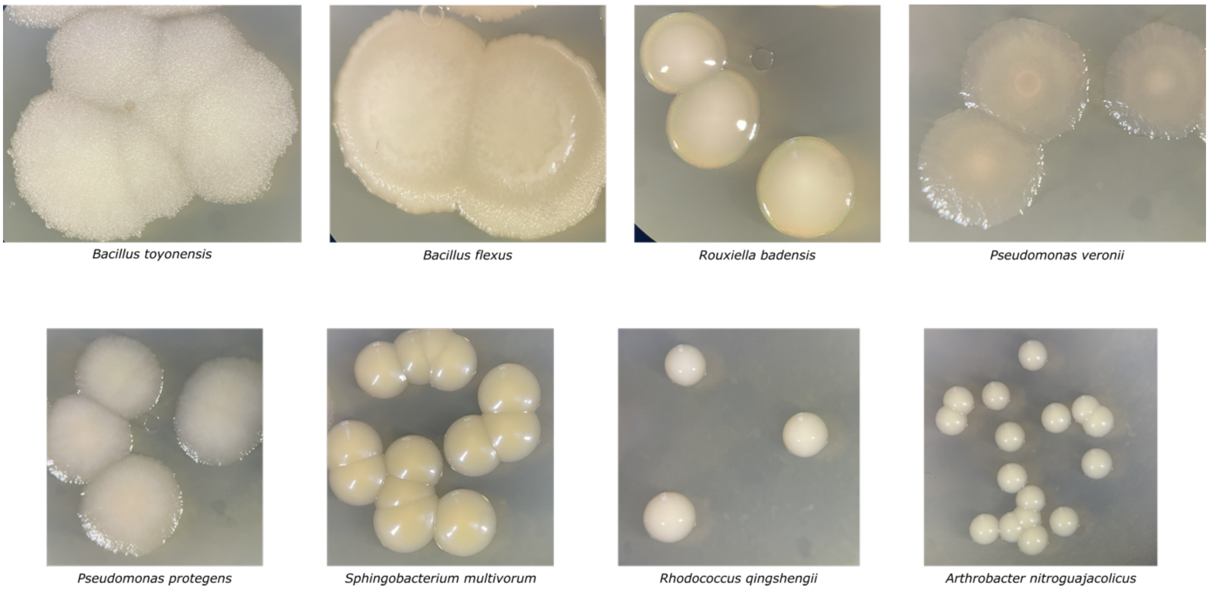

Supplementary Fig. 13: Colony morphologies of 8 *C. elegans* gut species. Each species was identified using size, color and specific distinct features. Bacterial colonies have many features like size, color, transparency, surface roughness/shininess, and granularity. Only some of those features are visible in the photos provided. For example, *Bacillus toyonensis* displays ‘white, granular and rough’ surface, while *Bacillus flexus* was identified as ‘large, yellow and rough’ colonies. *R. qingshengii* appears as ‘small, white and shiny’ colonies while *A. nitroguajacolicus* appears as “small, greenish and shiny colonies”. Similarly, other species were identifiable with distinct features. These differences allowed us to obtain the number of colony forming units (CFU) of different species even within our 8-species communities. Counting of colony forming units has compared to amplicon sequencing the advantage that absolute not only relative abundances could be obtained.

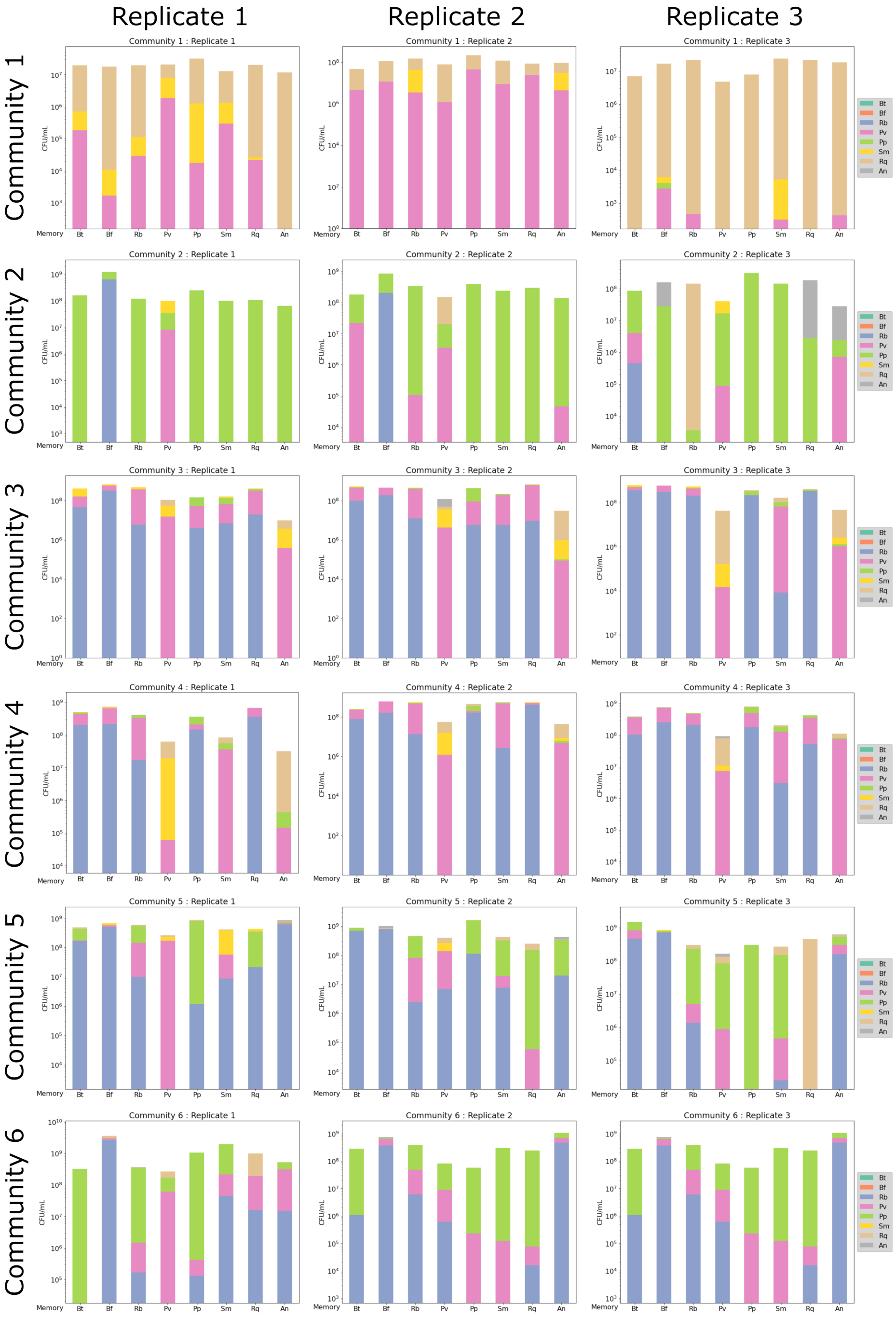

Supplementary Fig 14: Community assembly experiment on Day 5. Community composition on Day 5, determined from plating on NM agar. Each row shows a different initial community composition. Columns correspond to technical replicates. The bars in each plot show the development of initial communities in the externalized and collective memory of the species named on the x-axis. Within each bar, species are stacked linearly, whereas bars in bar plots are log-scaled, thereby maintaining relative distribution of species.

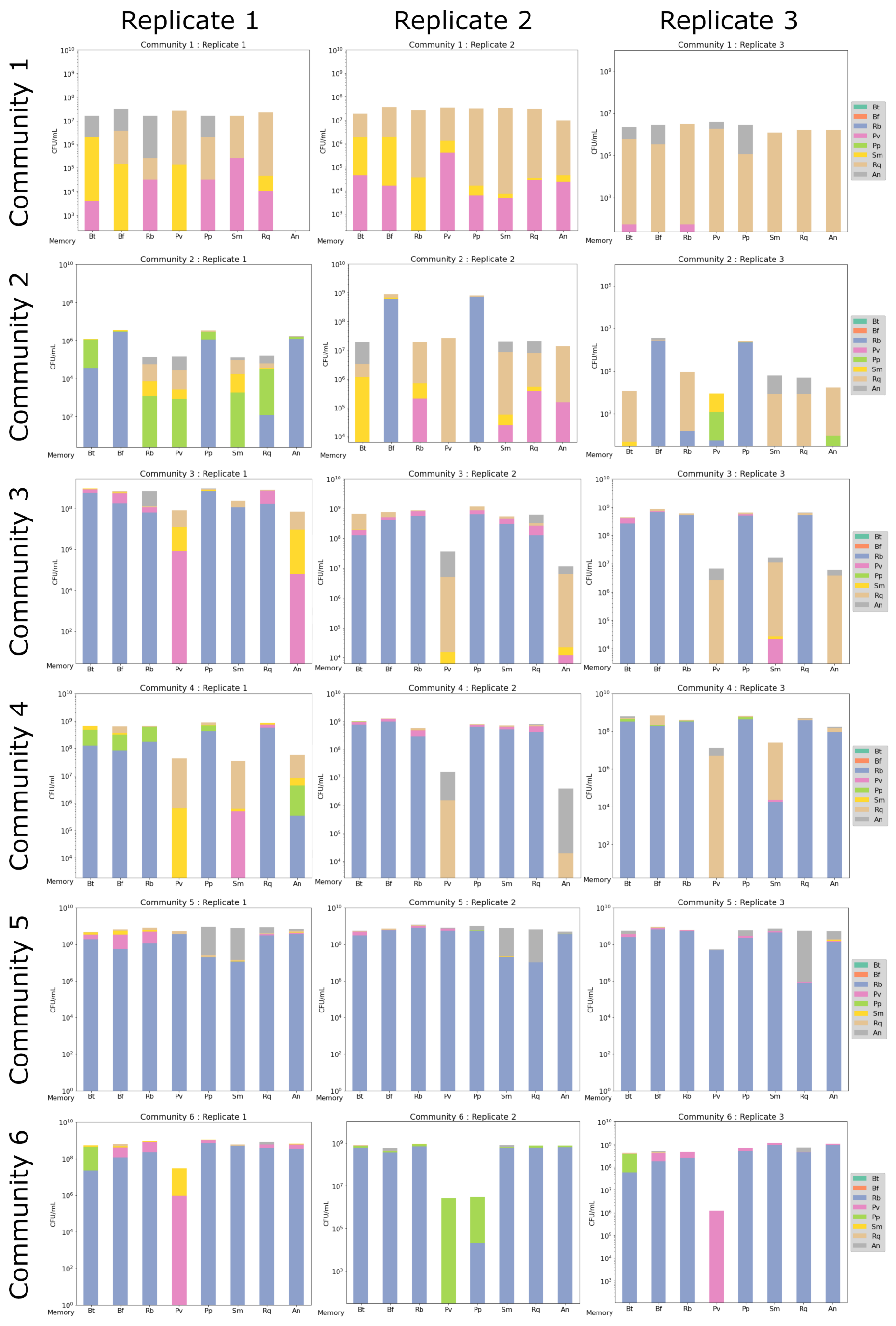

Supplementary Fig 15: Community assembly experiment on Day 10. Community composition on Day 10, determined from plating on NM agar. Each row shows a different initial community composition. Columns correspond to technical replicates. The bars in each plot show the development of initial communities in the memory of the species named on the x-axis. Within each bar, species are stacked linearly, while bars in bar plots are log-scaled, thereby maintaining relative distribution of species.

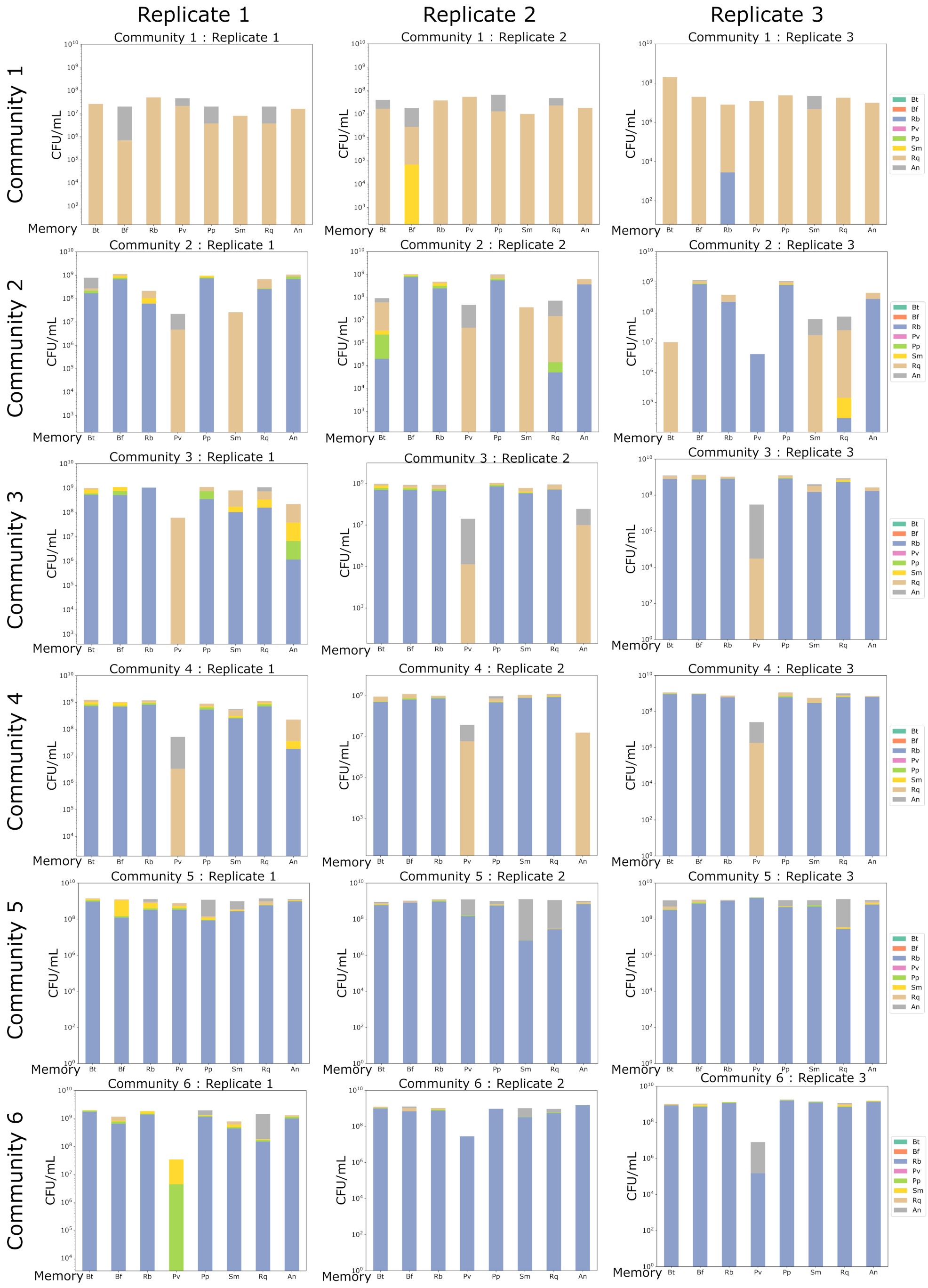

Supplementary Fig 16: Community assembly experiment on Day 12. Community composition on Day 12, determined from plating on NM agar. Each row shows a different initial community composition. Columns correspond to technical replicates. The bars in each plot show the development of initial communities in the memory of the species named on the x-axis. Within each bar, species are stacked linearly, while bars in bar plots are log-scaled, thereby maintaining relative distribution of species.

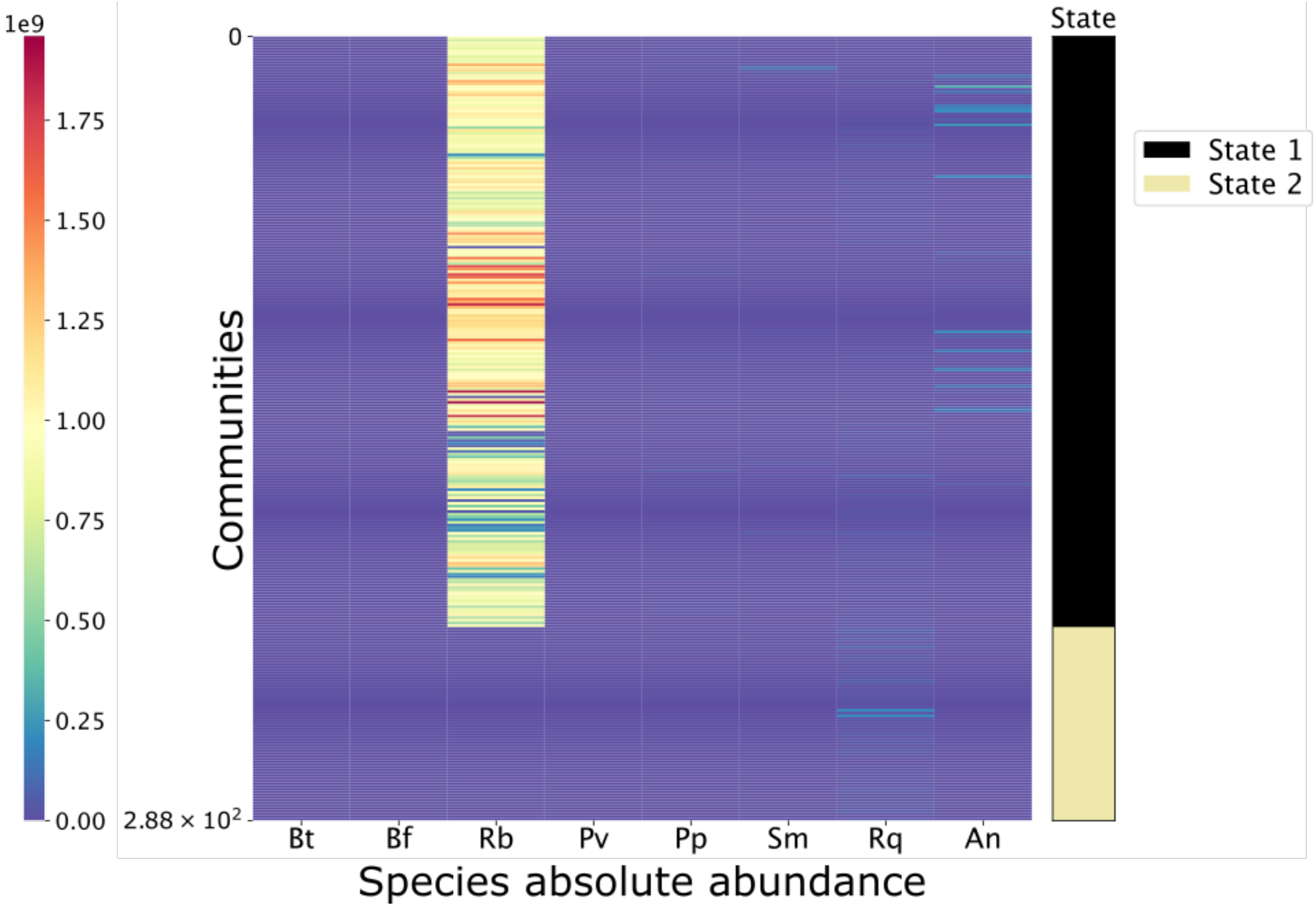

Supplementary Fig 17: 8-species communities assemble into two distinct states. The heatmap represents the absolute abundance of each species in the final community compositions of the initial 8 species. Each row represents the assembly of one community. The color bar represents state IDs derived from Hierarchical clustering (based on Bray-Curtis dissimilarities). State 1 communities are mostly dominated by *Rb*, while State 2 communities exhibit presence of *Rq, Sm, An, Pp* and *Rb,* but at a lower total cell density. Although 'symlog' is used for heatmap scaling, subdued tones of *Pp, Sm, Rq,* and *An* in state 2 are results of low absolute abundances of these species as compared to *Rb* in state 1 communities.

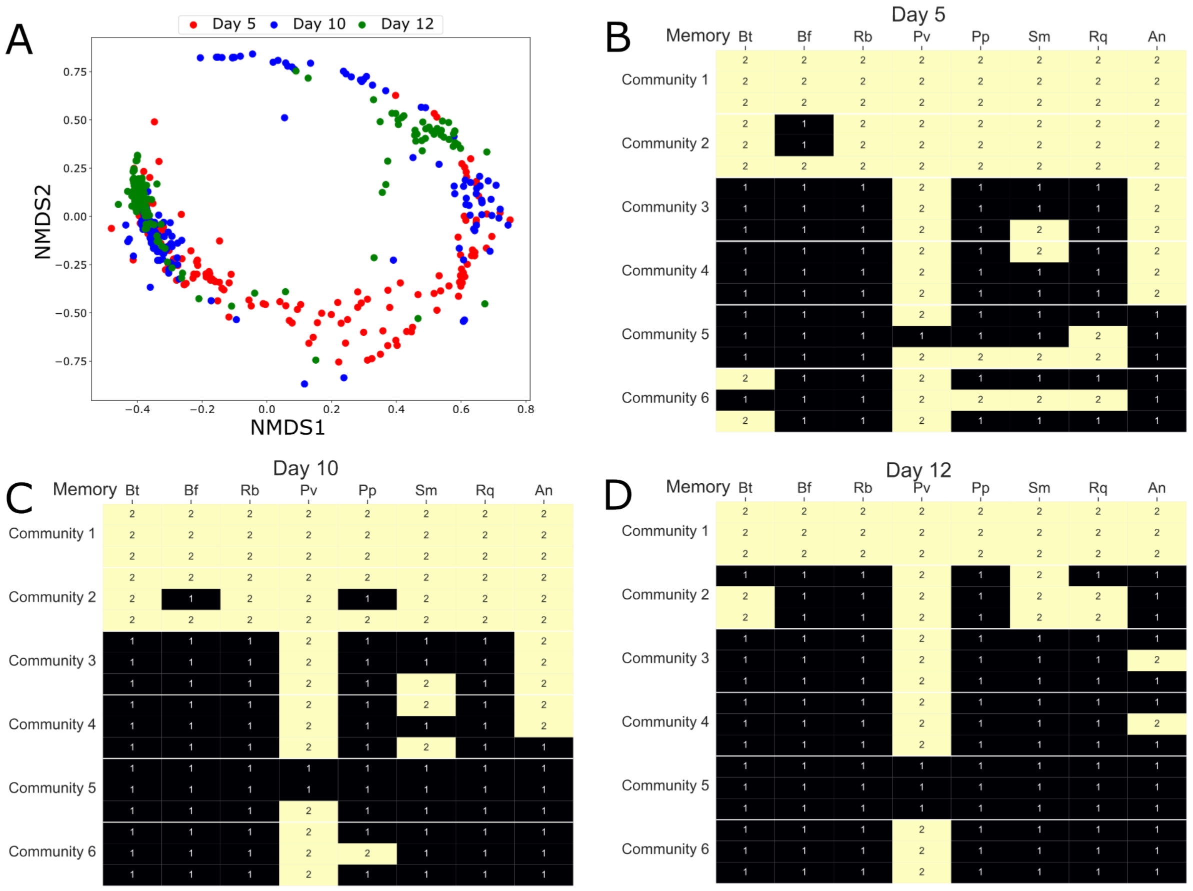

Supplementary Figure 18: Assemblies that start at different initial compositions converge towards two states over time. (A) To study the community assembly over time, community composition data of communities assembled in the presence of externalized memories from Day 5, Day 10 and Day 12 were combined and an NMDS plot was constructed from their Bray-Curtis dissimilarities (as in Fig. 4 and described in the Methods). Whereas on day 5 the communities are still rather dispersed in the NMDS plot, they converge towards two states on day 10 and even more day 12. The state membership of each assembling community based on clustering (see Supplementary Fig. 12C) is shown over time for all initial communities in different memories across three replicates each after 5 days **(B)**, 10 days **(C)** and 12 days **(D)**. Whereas for some conditions assemblies reach their final states already after 5 days (e.g., communities in the memory of *Pseudomonas veronii* (*Pv*)), other assemblies change their state membership even after 10 days (e.g., community 2 assemblies).

Supplementary Text:

Theoretical conditions for the appearance of externalized memory

For the appearance of the memory effect, two conditions have to be fulfilled:

1) Interactions have to be environmentally mediated

Externalized memory can lead to different interaction outcomes for the same mixing ratio of a set of N species. Such a memory cannot occur for direct interactions that are not mediated through the environment. In the case of direct interactions, the interaction outcomes of N species only depend on the abundances of these species themselves. Therefore, for given initial abundances of the N species the same interaction outcomes will be achieved (ignoring noise), because the system's dynamics only depend on those N variables and no other variables are present.

This is for example true for a Lotka-Volterra type system:

$$\frac{dN_i}{dt} = N_i \left(1 - \sum_j \alpha_{ij} N_j\right)$$

Where the dynamics of the species N_i only depend on the abundance of all species N_j.

Accordingly, for different outcomes for the same initial abundances of N species another variable has to be present, which in our case is the environment. The value of this environment could have been changed in the past by species activity, and thus, memory can occur.

2) Separation of timescales between the change in the environment and population densities

The change of the environmental variable has to be rather slow compared to the change in species abundance. This can be seen from the main text Fig. 3c but also directly obtained from the underlying model:

$$\frac{dN_i}{dt} = \Theta(1 - |p - p_o|)N_i - r_{dilution} N_i \qquad (1)$$

$$\frac{dp}{dt} = \sum_i k_i N_i - r_{dilution} p \qquad (2)$$

If the change of p is much faster than the change of N_i, p can be considered in a steady state (dp/dt=0), and thus from Equation 2 we obtain

$$p = \frac{\sum_i k_i N_i}{r_{dilution}}$$

which gives with equation (1)

$$\frac{dN_i}{dt} = \left[\Theta(1 - \left|\frac{\sum k_i N_i}{d} - p_o\right|) - r_{dilution}\right] N_i$$

Accordingly, the change of N_i does not depend on p anymore. Potential past changes of p will not affect the dynamics of N_i and, thus, the interaction outcomes. That means memory has no impact anymore.

Ecological concepts	Definitions
Legacy effect	"An indirect effect that persists for a long time period in the absence of the causal species, or after this species has ceased the causal activity[9]."
Priority effect	"Even subtle differences in species arrival history can cause large differences in the structure and function of communities, as effects are amplified over time and space via population growth and interactions. These are the effects termed priority effects, in which the effect of species on one another depends on the order in which they arrive at a site[77]."
Niche construction	"Organisms through their metabolism, their activities, and their choices, define, partly create, and partly destroy their own niches. We refer to these phenomena as 'niche construction[8]."
Historical contingency	"The effect of the order and timing of past events on community assembly. Events that cause historical contingency in community assembly can be either abiotic or biotic[77]."
Ecosystems engineering	"The ecosystems engineering concept focuses on how organisms physically change the abiotic environment and how this feeds back to the biota[78]."

Supplementary Table 1: Describing ecological concepts that are related to memory. We refer to the definitions of the ecological concepts as given in the according references. However, in many cases these concepts are defined differently across different works, and several variations and extensions exist.
